# *β*-(4-fluorobenzyl) Arteannuin B induced interaction of ATF-4 and C/EBPβ mediates the transition of breast cancer cells from autophagy to senescence

**DOI:** 10.3389/fonc.2022.1013500

**Published:** 2022-11-17

**Authors:** Khalid Bashir Mir, Mir Mohd Faheem, Syed Mudabir Ahmad, Javeed Ur Rasool, Tanzeeba Amin, Souneek Chakraborty, Madhulika Bhagat, Zabeer Ahmed, Asif Ali, Anindya Goswami

**Affiliations:** ^1^ Academy of Scientific & Innovative Research (AcSIR), Ghaziabad, India; ^2^ Pharmacology Division, Council of Scientific & Industrial Research (CSIR)-Indian Indian Institute of Integrative Medicine, Jammu, India; ^3^ School of Biotechnology, University of Jammu, Jammu, India; ^4^ Natural Product and Medicinal Chemistry Division, Council of Scientific & Industrial Research (CSIR)-Indian Institute of Integrative Medicine, Jammu, India; ^5^ Division of Medicinal and Process Chemistry, Council of Scientific & Industrial Research (CSIR)-Central Drug Research Institute, Lucknow, Uttar Pradesh, India

**Keywords:** arteannuin B, ATF-4, C/EBPβ, breast cancer, senescence, autophagy

## Abstract

ATF-4 is a master regulator of transcription of genes essential for cellular-adaptive function. In response to the quantum and duration of stress, ATF-4 diligently responds to both pro-apoptotic and pro-survival signals converging into either autophagy or apoptosis/senescence. Despite emerging cues implying a relationship between autophagy and senescence, how these two processes are controlled remains unknown. Herein, we demonstrate *β*-(4-fluorobenzyl) Arteannuin B (here after Arteannuin 09), a novel semisynthetic derivative of Arteannuin B, as a potent ER stress inducer leading to the consistent activation of ATF-4. Persistent ATF-4 expression at early time-points facilitates the autophagy program and consequently by upregulating p21 at later time-points, the signaling is shifted towards G_2_/M cell cycle arrest. As bZIP transcription factors including ATF-4 are obligate dimers, and because ATF-4 homodimers are not highly stable, we hypothesized that ATF-4 may induce p21 expression by physically interacting with another bZIP family member i.e., C/EBPβ. Our co-immunoprecipitation and co-localization studies demonstrated that ATF-4 is principally responsible for the autophagic potential of Arteannuin 09, while as, induction of both ATF-4 and C/EBPβ is indispensable for the p21 regulated-cell cycle arrest. Interestingly, inhibition of autophagy signaling switches the fate of Arteannuin 09 treated cells from senescence to apoptosis. Lastly, our data accomplished that Arteannuin 09 is a potent inhibitor of tumor growth and inducer of premature senescence *in vivo*.

## 1 Introduction

The choice between life and death is one of the crucial functions of cells building an organism. Adjustments to altered microenvironment by remodeling cellular components to achieve homeostasis result in survival, while failure to adapt to stressful conditions leads to cell death. Autophagy may be part of these mechanisms, but how autophagy empowers the survival of tumor cells under stress warrants more detailed research. Under stress, cancer cells set off a “call” whether they can defy the nature and intensity of stress by promoting autophagy, go for cell cycle arrest, or capitulate to apoptosis due to an unbearable microenvironment. Autophagy, a highly dynamic and conservative multistep process, recycles cellular components to sustain survivability (1).

The essential organelle endoplasmic reticulum (ER) is involved in maintaining proper protein folding nature as well as calcium homeostasis (2, 3). Under certain stressful stimuli like accumulation of reactive oxygen species (ROS), acid-base imbalance, hypoxia, and nutrient deprivation, protein-folding capacity in the ER is perturbed, leading to the expansion of misfolded proteins causing ER stress (4). The evolutionary conserved unfolded protein response (UPR), protective in nature, resolves stress and dysfunction in ER. Initially, prolong ER stress leads to abrogation of global translation along with transcriptional upregulation of protein chaperones and hundreds of other genes involved in global proteostasis controlling protein refolding and processing (5). Even though autophagy and ER stress can function independently, they share several common attributes, including protecting cells by mitigating stress and inducing cell death under certain conditions (6). UPR signaling induced by mild to moderate stress is a compensatory mechanism for switching into adaptation programs (7). Data from several studies indicate the dependency of numerous tumors on autophagy, as they use autophagy as a nutrient replenishment mechanism to survive stressful conditions (8).

An innate response, senescence in cancer cells against the stress, is to avoid the proliferation of potentially malignant cells in an irreversible manner (9). Autophagy, on the other hand is reported to be closely associated with cellular senescence, and suppression of autophagy impedes cellular senescence in mitotic human diploid cells (10). Strikingly, cancer cells, not the normal cells, perpetuate by escaping cellular senescence (11).

Among the major proteins involved in ER stress, ATF-4 (Activating Transcription Factor 4), a ubiquitously expressed member of the ATF/CREB transcription factor family of basic leucine zipper (bZIP) superfamily, plays the role of the master regulator in stress management. ATF-4 modulates the transcription of a group of downstream target genes involved in cell survival, apoptosis, autophagy, and senescence (12–14). The eventual outcome following ATF-4 activation is context-dependent, influenced by the cell type and the nature of the stimulation (15). Interestingly, recent reports underscore that the activation of ER stress and hypoxia induce the transcription of *Map1lc3b*, an essential autophagy gene in ATF-4 dependent manner (16, 17). C/EBPβ (CCAAT/enhancer-binding protein β) belongs to basic leucine zipper (bZIP) family transcription factor and is known to regulate proliferation in several target tissues, including adipose tissue, liver and mammary glands (18). In order to function properly ATF-4 must dimerize to activate genes (19). As ATF-4 homodimers are not stable (20), consequently ATF-4 has a strong tendency to physically interact and form heterodimers with other bZIP family transcription factors including C/EBPβ (21).

The classical role of p21 as a tumor-suppressor and in regulating G_2_/M cell cycle arrest is a well-established fact (22). Although known for its growth repressive functions, cyclin-dependent kinase inhibitor (CDKI) p21 also functions in an anti-apoptotic manner (23). The survival role of p21 may contribute to ER stress-mediated activation of ATF-4 (24). p21 has been reported to play pivotal roles in triggering principal genes responsible for senescence and aging (25, 26).

The current study aims to investigate autophagy to senescence switching by a natural product and anti-cancer compound Arteannuin 09 and its effect on cellular homeostasis in breast cancer cells. *β*-(4-fluorobenzyl) arteannuin B (Arteannuin 09) is a semisynthetic derivative of the natural product arteannuin B derived from *Artemisia annua*, which exhibits antiproliferative activity in various breast and lung cancer cell lines (27). Structurally, Arteannuin 09 contains a rigid cadinane sesquiterpene unit with significant stereochemical diversity. This report unveils the role of ATF-4 and C/EBPβ in autophagy to senescence switching by upregulating the cell cycle protein p21.

## 2 Materials and methods

### 2.1 Reagents and antibodies

Arteannuin 09 (previously identified as 3i) was synthesized through coupling with arylboroic acid (27). ^1^H and ^13^C, DEPT NMR Spectra and HPLC purity data of Arteannuin 09 is given in **Supplementary Section 1.3, 1.4**. The breast cancer cell lines, MCF-7 and MDA-MB-231 used in this study were cultured in RPMI-1640 and Leibovitz’s L-15 medium respectively supplemented with 10% fetal bovine serum and 1% penicillin/streptomycin in a humidified CO_2_ incubator with 5% CO_2_. 4T1 (Mouse mammary carcinoma) cells were a kind gift from Dr Avinash Bajaj, Regional Center for Biotechnology, New Delhi, India and were cultured in the RPMI-1640 medium. The list of material and antibodies and their dilutions used in this study are provided in **Supplementary Tables 1**, **2**.

### 2.2 Plasmids and transfections

pEGFP-LC3 (human) was a gift from Toren Finkel (Addgene plasmid# 24920; http://n2t.net/addgene:24920; RRID: Addgene_24920). pEGFP was a gift from Koen Venken (Addgene plasmid# 165830; http://n2t.net/addgene:165830; RRID: Addgene_165830). Transient transfections were carried out using lipofectamine 3000 transfection kit according to the manufacturer’s protocol (Invitrogen, Carlsbad, CA).

### 2.3 siRNA transfection

Transient knockdown of AMPK, p21 and C/EBPβ was achieved by transfecting cells with respective siRNAs (sequences given in **Supplementary Table 3**). Human AMPK (PRKAA1) siRNA (AM16708) was procured from Invitrogen. p21 (CDKN1A) mission esiRNA (EHU003861) was procured from Sigma Aldrich; and C/EBPβ siRNA (h) (SR300760) was procured from OriGene Technologies, Inc. Briefly, MCF-7 and MDA-MB-231 cells were seeded in 60 mm dishes and transiently transfected after 24 h with scramble/siRNA AMPK, scramble/siRNA p21 and scramble/siRNA C/EBP β using Lipofectamine 3000 reagent. After 24 h, the cells were subjected to treatment with Arteannuin 09 and accordingly harvested. Cell lysates were prepared to conduct Western blot analysis.

### 2.4 SA-β-gal staining assay

Cells (0.3 × 10^6^) were seeded in six-well plates and treated with vehicle or Arteannuin 09 (1 µM) for 48 h. The procedure for SA-β-gal staining was followed as described previously (28). Accordingly, stained cells were thoroughly washed and air dried in dark. Cells were then observed under bright-field microscope (NIKON, Nikon Corporation, Chiyoda-ku, Tokyo, Japan) for the SA-β-gal-positive cells and images were captured at 20 x magnification.

### 2.5 SAHF detection assay

The detection for senescence-associated heterochromatin foci (SAHF) was carried out as previously described (29). About 1 × 10^5^ MCF-7 and MDA-MB-231 cells were seeded on cover slips and treated with Arteannuin 09, positive control Doxorubicin or Vehicle for five days. Cells were subsequently washed with PBS and fixed with 4% paraformaldehyde. Cells were again washed with PBS and stained with DAPI containing mounting media. Images were captured using 20 x objective on Floid cell imaging station (Invitrogen).

### 2.6 Western blot analysis

Western blotting was performed as described previously (30). Following the respective treatments and transfections (see figure legends), cell lysates were prepared with lysis buffer containing protease and phosphatase inhibitors. Subsequently, lysates were centrifuged at 12000 rpm for 10 min at 4°C. Standard Bradford method was employed to determine protein concentration. Equal amount of protein (30-40 µg) was applied for SDS-PAGE, separated proteins were accordingly transferred to PVDF membranes, blocked with 5.0% (w/v) BSA and subsequently probed with primary antibodies (for 4 h at room temperature or overnight at 4°C). Blots were then washed with TBST and probed with species-specific secondary antibodies. Blots were again washed thoroughly and immunoreactive protein bands detected by using western Bright ECL HRP substrate (Advansta) and exposed over the CL-XPosure film (Thermo Scientific).

### 2.7 Preparation of cytoplasmic and nuclear extracts

Nuclear and cytoplasmic fractions were obtained according to the previously described procedure by our group (31). Arteannuin 09 treated MCF-7 cells were washed with ice cold PBS and centrifuged.Cell pellets obtained were then treated with ice cold hypertonic and hypotonic buffers to separate out the nuclear and cytosolic extracts. Western blotting was employed to analyse the expression of various proteins.

### 2.8 Immunocytochemistry

For p21 and p-AMPK protein immunocytochemistry, MCF-7 and MDA-MB-231 cells were seeded on cover slips at a density of 0.5 × 10^6^ cells per well. Post transfection with or without si-RNA and/or treatment with Arteannuin 09, permanent mounts of the coverslips were prepared by following the previously published protocol (32). The antibodies used and their working dilutions are provided in **Supplementary Table S2**. The prepared slides were then analyzed under Floid Cell Imaging Station (Invitrogen) at × 20 magnification.

### 2.9 Cell cycle analysis

MCF-7 and MDA-MB-231 cells were seeded at 0.3 × 10^6^ cells per well on 35-mm dishes and incubated overnight at 37°C and 5% CO_2_. Following transfections or treatments with Arteannuin 09, the cells were incubated for set time points. At the end of incubation period, adherent cells were trypsinized, centrifuged at 1500 rpm for 5 min, washed thrice with ice-cold PBS and fixed with ice-cold 70% ethanol (v/v) for 1 h at 4°C. Cells were again washed with PBS and incubated with 50 μg/ml of Propidium iodide (PI) and 200 μg/ml of RNase-A in PBS at RT for 30 min in dark. Acquisition was performed on BD flow cytometer and data were analyzed using FlowJo software (10.7.2).

### 2.10 Annexin V/PI staining

For quantification of apoptotic cells Annexin V-FITC Apoptosis Detection Kit (Sigma-Aldrich) was used. Briefly, after the incubation with Arteannuin 09 for indicated time, MCF-7 cells were trypsinized and externalized phosphatidylserine was labeled with annexin-V-FITC-conjugated for 15 min on ice. Prior to FACS analysis Propidium iodide (PI, 1 μg/mL) was added for 10 min. Stained cells were analyzed by BD C6 flow cytometer.

### 2.11 Co-immunoprecipitation assay

ATF-4 and C/EBPβ immunoprecipitation experiments were performed by following the previously described protocol (33). Briefly, MCF-7 cells (2.5 × 10^6^) were seeded in 90 mm dishes followed by Arteannuin 09 treatment for either 6 h or 48 h. Post treatment, cells were harvested and pelleted. RIPA buffer was used for cell lysis and then lysates were subjected to pre-clearance with 25 μL of Protein-G Plus agarose beads (SantaCruz, USA). Immunoprecipitation was performed with 5 μg of antibody conjugated to 50 μL of Protein-G Plus agarose beads. Rabbit IgG was used as a control antibody for pull-down experiments. Subsequently, immunoprecipitates were washed with RIPA buffer and subjected to immunoblotting. IP clean blot HRP–tagged secondary antibody (Thermo Scientific, USA) was employed for detection purposes and to eliminate heavy and light chain immunoglobulin interference.

### 2.12 Acridine orange staining

AO staining is used to detect and quantify acid vesicular organelles. MCF-7 cells were cultured in presence or absence of Arteannuin 09 for 6 h and after treatment, cells were incubated with AO (1 μg/mL) for 15 min, trypsinized and followed by washing with PBS. The bright red fluorescence was detected and analyzed in the fluorescence microscope (Floid Cell Imaging Station, Invitrogen).

### 2.13 *In vivo* study

In accordance with the institutional guidelines proper care was taken to maintain the animals in good health and aseptic conditions were maintained through out the study. To evaluate the *in vivo* antitumor activity of Arteannuin 09, healthy female Balb/c mice (5-7 weeks old, body weight 25–30 g) were taken. 1.5 × 10^6^ 4T1 mouse mammary carcinoma cells were suspended in 100 µL of serum-free RPMI medium and injected subcutaneously into the mammary fat pad of each mouse around the second right mammary gland. When the palpable tumors developed after one week of tumor initiation, the animals (n=8) were randomized into three groups. The animals were injected with vehicle (1% DMSO + 5% Tween-80 + 24% PEG-400 + 70% normal saline v/v) or Arteannuin 09 (25 mg/kg b.w.) or Doxorubicin as positive control (10 mg/kg b.w.) intraperitoneally on each alternative day for two weeks. The dose for Arteannuin 09 was choosen on the basis of dose response study given in **Supplementary Section 2.5**, while as the dose for doxorubicin at 10 mg/kg b.w. was choosen on the basis of literature survey (34). Tumor sizes were measured on alternate days and body weight was recorded twice a week. Mice were sacrificed by CO_2_ euthanasia followed by cervical dislocation in a humane way after 15 days from the initiation of treatment, and tumors were dissected out carefully for evaluation.

### 2.14 Statistical analysis

Data are expressed as mean ± SEM of at least three independent experiments. GraphPad Prism software (Version 5.0) was used for statistical purposes. Comparisons between two groups were conducted using one-way ANOVA followed by Bonferroni post-tests. *P < 0.05. **P< 0.01. ***P < 0.001 values were assigned significance.

## 3 Results

### 3.1 Arteannuin 09 induced autophagy is mediated through ER stress response axis

**Figure 1:**
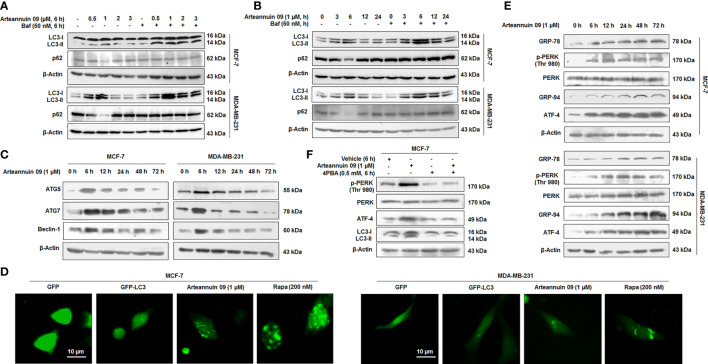
Arteannuin 09 induces ER stress-mediated autophagy **(A)** Immunoblotting analysis of LC3-I/II and p62 showing autophagic flux after treatment with indicated concentrations of Arteannuin 09 in the presence and absence of 50 nM Bafilomycin for 6 hours in MCF-7 and MDA-MB-231 cell lines. β-actin was taken as the loading control. **(B)** Immunoblotting analysis of LC3-I/II and p62 after treatment with 1 µM Arteannuin 09 for different time points (0-24 h) in the presence and absence of 50 nM Bafilomycin in MCF-7 and MDA-MB-231 cell lines. β-actin was taken as the loading control. **(C)** MCF-7 and MDA-MB-231 cells were treated with 1 µM Arteannuin 09 for indicated time points and analyzed for the expression of ATG5, ATG7, and Beclin-1 through immunoblotting. **(D)** Fluorescence images of Puncta formation by LC3-I/II in MCF-7 and MDA-MB-231 cells after transient transfection with GFP/GFP-LC3 and treatment with 1 µM Arteannuin 09 for 6 h, magnification - 20X, scale bar - 10 µm. Rapamycin was taken as a positive control. **(E)** Immunoblotting analysis of ER stress response proteins GRP-78, p-PERK, PERK, GRP-94, and ATF-4 after treatment with 1 µM concentration of Arteannuin 09 for different time points (0-72 h). **(F)** Immunoblotting analysis of p-PERK, PERK, ATF-4, and LC3-I/II after treatment with Arteannuin 09 and 4PBA alone or in combination for 6 hours in MCF-7 cells. β-actin was taken as the loading control. Densitometry analysis given in **Supplementary Information (section 2.6)**. Each experiment was carried out in triplicates (N=3).

We have previously reported a fluorobenzyl derivative of Arteannuin B, Arteannuin 09 (*β*-4-fluorobenzyl arteannuin B), as anti-cancer agent, particularly in breast cancer cell lines: MCF-7 and MDA-MB-231 (27). Based on our preliminary study (Data not shown), we were interested in screening active Arteannuin B derivatives for their autophagy-inducing potential. Among all the derivatives of Arteannuin B, Arteannuin 09 was identified as the most potent autophagy inducer (**Supplementary Figure 2.1**). Dose and time-dependent studies were carried out to assess the effect of Arteannuin 09 on autophagy flux (**Figures 1A, B**). MCF-7 and MDA-MB-231 cells were treated with Arteannuin 09 in the presence or absence of Bafilomycin A1 (BAF). BAF blocks the fusion between autophagosomes and lysosomes (35). Immunoblotting of MCF-7 and MDA-MB-231 cells showed steady turnover of LC3B-I to autophagosome-associated LC3B-II, a conventional marker of autophagosome assembly. Moreover, to detect the effect of Arteannuin 09 on autophagic flux, the expression of SQSTM1 (sequestosome 1), a selective substrate of autophagy, was analyzed (36). The level of SQSTM1 reduced sharply at 6 hours in both the cell lines upon the treatment with Arteannuin 09, suggesting an enhanced autophagic flux (**Figures 1A, B**). Based on these experiments, the optimum autophagic flux was observed at 6 h time-point with 1µM dose of Arteannuin 09. Additionally, the effect of Arteannuin 09 treatment on the major autophagy-related proteins ATG5, ATG7, and Beclin-1 was evaluated by WB analysis. Interestingly, Arteannuin 09 augmented the levels of ATG5, ATG7, and Beclin-1 in MCF-7 and MDA-MB-231 cells at 6 h time-point (**Figure 1C**). Off note the elevation in ATG5, ATG7 and Beclin-1 at 6 h time point was concomitant with the conversion of LC3B-I to LC3B-II along with ablation of p62 expression. Furthermore, to validate our immunoblotting results, we performed LC3B puncta assay by fluorescence imaging showing pronounced puncta formation in both MCF-7 and MDA-MB-231 cells when treated with Arteannuin 09 (1 µM) (**Figure 1D**).

Numerous studies have shown Endoplasmic Reticulum (ER) stress as a potent autophagy inducer (37–39). ER stress activates autophagy *via* unfolded protein response (UPR)-mediated up-regulation of components of the autophagy machinery. Our previous report identified GRP-78 as a putative target of Arteannuin 09 (27). In order to confirm whether Arteannuin 09 induced autophagy is linked with ER stress, we treated MCF-7 and MDA-MB-231 cells with 1 µM Arteannuin 09 and performed immunoblotting for vital ER stress markers (**Figure 1E**). Indeed we found a prominent induction of ER stress markers including p-PERK (Thr 980), GRP-78, GRP-94, from 6 h to 72 h. We also sought to investigate the effect of Arteannuin 09 treatment on ATF-4, a principal stress-induced transcription factor responsible for activating wide array of stress adaptive genes. Pertinently, consistent activation of ATF-4 was observed along with ER stress markers from 6 h onwards (**Figure 1E**). Furthermore, to confirm the essentiality of ER stress induction in Arteannuin 09 mediated autophagy, we co-treated MCF-7 cells with 4-PBA (a chemical chaperone that relieves ER stress) along with 1 µM Arteannuin 09. Alleviation of ER stress by 4-PBA resulted in a decrease in Arteannuin 09 mediated autophagy (**Figure 1F**). Collectively, our results unravel Arteannuin 09 as a dynamic autophagy inducer, primarily mediated through ER stress response.

### 3.2 AMPK is essential to ameliorate Arteannuin 09 induced autophagy

**Figure 2:**
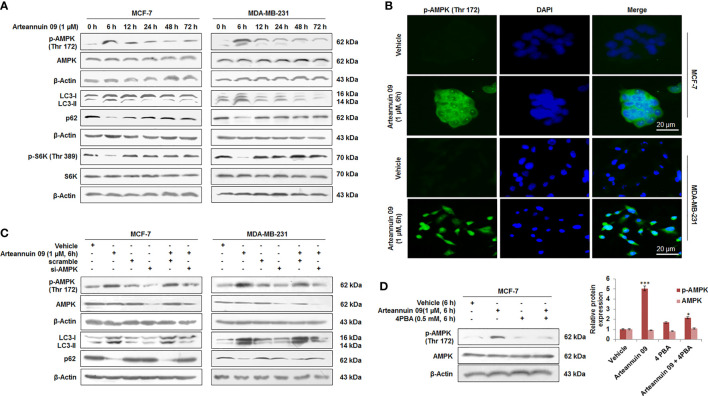
Arteannuin 09 induces autophagy by up-regulating p-AMPK **(A)** MCF-7 and MDA-MB-231 cells subjected to Immunoblotting after the time dependent treatment of Arteannuin 09 (1 µM) for the expression of p-AMPK, LC3-I/II, p62, and p-S6K. β-actin was taken as the loading control. **(B)** Immuno-cytochemistry detection of p-AMPK carried out in MCF-7 and MDA-MB-231 cells after treatment of indicated concentrations of Arteannuin 09, N=3. Magnification 20X, scale bar–20 µm. **(C)** Immunoblots portraying the expression of p-AMPK, LC3-I/II and p62 in MCF-7 and MDA-MB-231 cells after Arteannuin 09 (1 µM) and si-AMPK transfection. β-actin was taken as the loading control. **(D)** Immunoblotting analysis of p-AMPK after treatment with Arteannuin 09 and 4PBA alone or in combination for 6 hours in MCF-7 cells. Densitometry analysis is given in **Supplementary Information** (section 2.6). Each experiment was carried out in triplicates (N=3) and results are expressed as mean± sd ***p < 0.001, *p <0.05.

To investigate the mechanisms of Arteannuin 09 induced autophagy, we studied the effects of arteannuin 09 on components of the mTOR pathway, including the AMP-activated protein kinase (AMPK) which regulates mTOR signaling. AMPK is the pivotal energy sensing protein kinase in almost all eukaryotic cells (40). It inhibits anabolic processes and promotes catabolic processes (41). Arteannuin 09 was found to induce the pAMPK-mTOR-S6K pathway, which plays a vital role in autophagy and protein translation. To explore molecular events involved in autophagy induced by Arteannuin 09, we set up a time-pulse (0 h to 72 h) experiment in MCF-7 and MDA-MB-231 cells treated with 1 µM of Arteannuin 09. The results showed that MCF-7 and MDA-MB-231 cells treated with Arteannuin 09 displayed a robust increase in p-AMPK and significant inhibition of p-S6K at the autophagy inducing time-point (6 h) (**Figure 2A**). Furthermore, immunocytochemistry of p-AMPK was carried out resulting in a robust increase in p-AMPK level as early as 6 h time point in MCF-7 and MDA-MB-231 cells following Arteannuin 09 treatment compared to vehicle at (**Figure 2B**).

The role of p-AMPK in Arteannuin 09 induced autophagy was validated through transient knock down of AMPK in MCF-7 and MDA-MB-231 cells employing si-AMPK. Notably, in the AMPK knockdown condition, Arteannuin 09 failed to induce any LC3B-I to LC3B-II conversion along with the reduction in p62 level suggesting that AMPK is indispensable for autophagy induced by Arteannuin 09 (**Figure 2C**). Additionally, in the presence of 4PBA (a chemical chaperone that relieves ER stress), it failed to activate AMPK, thereby, confirming ER stress as a prerequisite for Arteannuin 09 induced AMPK activation (**Figure 2D**). Taken together, these results suggest a vital role of p-AMPK in Arteannuin 09 mediated autophagy induction.

### 3.3 Arteannuin 09 stalls G_2_/M phase coupled with p21 mediated senescence induction at later time points

**Figure 3:**
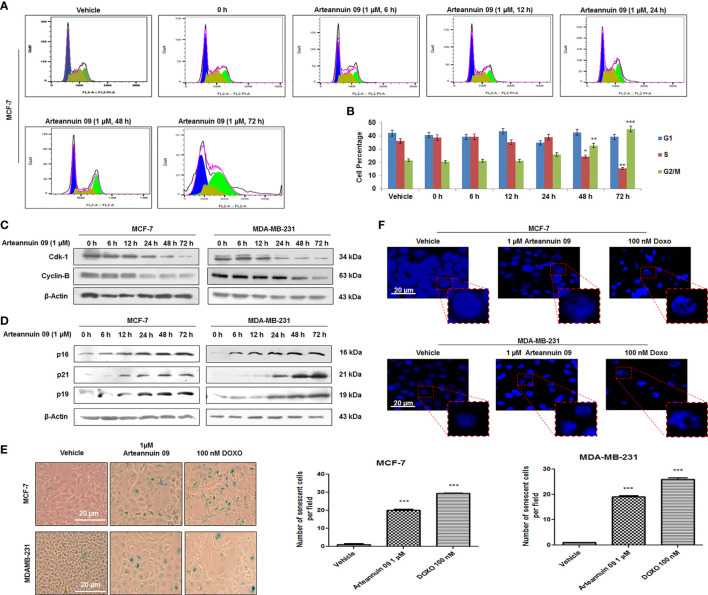
Arteannuin 09 induces premature-senescence in breast cancer cells. **(A, B)** Cell cycle (PI staining) analysis of cells treated with Arteannuin 09 in a time-dependent manner (0-72 h), N=3. Cell cycle analysis was performed with FlowJo software by employing the Watson (pragmatic) model. Black line represents actual cell population, pink line represents how accurately the model fits the cell population. Blue color represents G_1_, olive S phase and parrot green G_2_/M phase. **(C)** Immunoblotting analysis of Cdk-1 and Cyclin-B proteins in MCF-7 and MDA-MB-231 cells after Arteannuin 09 (1 µM) treatment in time-dependent manner. **(D)** Immunoblotting analysis of senescence specific markers *viz.* p16, p21, and p19 in MCF-7 and MDA-MB-231 cells. β-actin was taken as the loading control. **(E)** MCF-7 and MDA-MB-231 cells were treated with Arteannuin 09 (1 µM) and doxorubicin (100 nM) and incubated with X-gal for 48 h post treatment for analysis of SA-β-gal activity, N=3. Photo micrographs were taken under bright-filed microscope-Nikon Eclipse 200 at 20X magnification, scale bar – 20 µm. Bar graph representing the SA-β-gal positive cells in MCF-7 and MDA-MB-231 cells. **(F)** Observation of DAPI stained MCF-7 and MDA-MB-231 cells for SAHF after Arteannuin 09 (1 µM) treatment under Floid Cell Imaging Station at 20X magnification, N=3. The insets clearly show the formation of heterochromatin foci. Densitometry analysis given in **Supplementary Information** (section 2.6). Each experiment was carried out in triplicates (N=3) and results are expressed as mean± sd ***p < 0.001, **p < 0.01, *p <0.05.

While autophagy influx can determine alternative cellular fates by facilitating other homeostatic stress responses such as cell cycle arrest and premature senescence, the functional interlinks remain yet to be deciphered. In the previous sections, we have demonstrated that Arteannuin 09 mediated autophagy induction occurs at an early time-point (6 h) of treatment. Rationally, we sought to explore the cell fate with prolong exposure (24 h to 72 h) of breast cancer cells with Arteannuin 09. Intriguingly, FACS data demonstrated that Arteannuin 09 (1 μM) treatment (for 48 and 72 h) arrested MCF-7 cells in the G_2_/M phase (32.8% and 45.2%, respectively) compared to the vehicle-treated cells (G_2_/M = 21.71%) (**Figures 3A, B**). Induction of the G_2_/M checkpoint also led to the suppression of cyclin-dependent kinase 1 (Cdk-1) and Cyclin-B, thereby leading to loss of proliferating capacity (22). Similarly, Arteannuin 09 (1 μM) treatment resulted in the abrogation of Cdk-1 and Cyclin-B expression post 24 h time-point (**Figure 3C**).

The involvement of p21 in G_2_/M regulation and the association of prolonged G_2_ arrest with senescence phenotype are emerging field of research. The expression of another Cyclin-dependent kinase inhibitor, p16^INK4a^, together with β-galactosidase staining, is considered a hallmark of senescence (42–44). Correspondingly, our immunoblotting results showed a robust increase in p21 expression along with the upregulation of p16 and p19, in MCF-7 and MDA-MB-231 cells particularly from 24 h onwards, (**Figure 3D**). In addition immunocytochemistry in MDA-MB-231 cells showed a robust increase in the expression of p21 at 48 h compared to 6 h post Arteannuin 09 treatment, (**Supplementary Figure 2.2**) To check the ability of Arteannuin 09 triggering premature senescence, we carried out SA-β-gal activity assays in MCF-7 and MDA-MB-231 cells following treatment with indicated concentrations of Arteannuin 09 along with Doxorubicin as a positive control. Strikingly, we observed a significant increase in SA-β-gal positive cells (95.23% in MCF-7 and 94.73% in MDAMB-231) and characteristic senescent features (flattened cellular morphology and bluish nuclear stains), distinctly visible in Arteannuin 09 treated cell lines compared to the vehicle-treated cells (**Figure 3E**). We also observed another signature of senescence-SAHF formation following Arteannuin 09/Doxorubicin treatment demonstrating appearance of a characteristic beaded nucleus. In contrast, vehicle-treated cells were devoid of such beaded nuclear phenotype (**Figure 3F**). These combined results firmly suggested Arteannuin 09 as a potential inducer of premature senescence by arresting cells at the G_2_/M checkpoint and activating p21 at later time points.

### 3.4 ATF-4 is a key upstream transcription factor mediating Arteannuin 09 effects– initiating from autophagy induction to termination into senescence cascade

**Figure 4:**
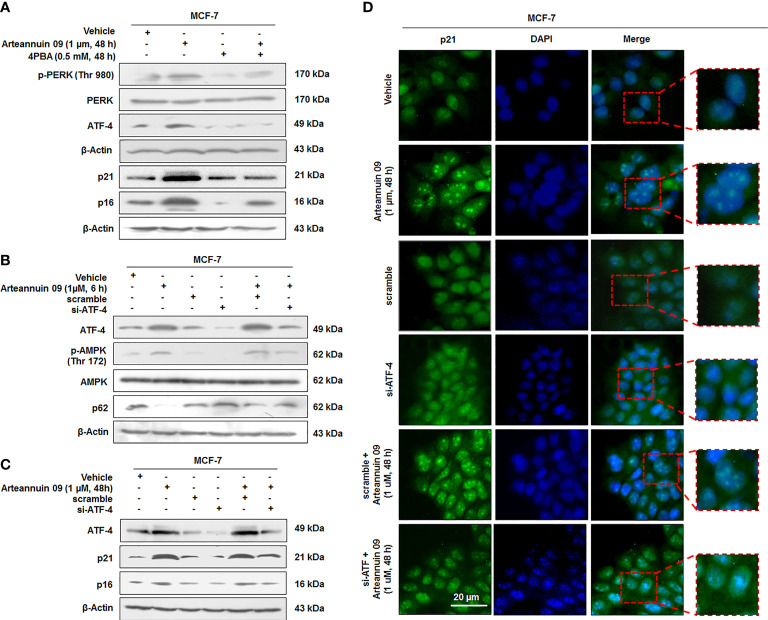
ATF-4 is central in mediating switching from Autophagy to Cell cycle arrest **(A)** Immunoblotting analysis of ER stress proteins p-PERK (Thr 980), and ATF-4 and senescence markers p21 and p16 in presence and absence of Arteannuin 09 (1 µM) and 4PBA (0.5 mM) at 48 h time point. β-actin was taken as the loading control. **(B, C)** Immunoblotting analysis of MCF-7 cell for the expression of ATF-4, p-AMPK, p62, p21 and p16 (autophagy to senescence switching) after si-RNA mediated knockdown of ATF-4 and Arteannuin 09 (1 µM) treatment for 6 h and 48 h respectively. β-actin was taken as the loading control. **(D)** Immunocytochemistry analysis of p21 in MCF-7 cells transfected with scramble, si-ATF-4, or treated with vehicle, Arteannuin 09 (1 µM), scramble plus Arteannuin 09 for 48 h. Magnification 20X, scale bar – 20 µm. Densitometry analysis given in **Supplementary Information** (section 2.6). Each experiment was carried out in triplicates (N=3).

Based on our findings in the earlier sections, we hypothesized that Arteannuin 09 induced ER stress might be the cause of the transition from autophagy to senescence. To confirm this, we co-treated cells with Arteannuin 09 plus 4-PBA (ER stress reliever) and the results implied decelerated ER stress markers along with ATF-4 levels and a concomitant diminished expression of senescence markers at 48 h time point (**Figure 4A**). Notably, ATF-4 controls the transcription of vital genes essential for adaptive functions (45, 46). Next, we were keen to investigate whether siRNA-mediated inhibition of ATF-4 could have any effects on Arteannuin 09 mediated senescence switching. Thus, MCF-7 cells were transfected with scramble and si-ATF-4, succeeded by treatment with 1 µM Arteannuin 09 for either 6 h (autophagic induction) or 48 h (senescence induction). Strikingly, Arteannuin 09 treatment (at 6 h) showed a robust decrease in p-AMPK and failed to reduce autophagy marker p62 in the si-ATF-4 condition, signifying the inability of Arteannuin 09 in inducing autophagy in ATF-4 transiently knocked-down MCF-7 cells. Of note, a simultaneous reduction in p21 and p16 expression was also achieved in si-ATF-4 plus Arteannuin 09 (at 48 h) treated MCF-7 cells, suggesting the failure of Arteannuin 09 in inducing senescence. (**Figures 4B, C**). Furthermore, immunocytochemistry was carried out to validate these above results. Thenceforth, MCF-7 cells were transfected with the same conditions as above and treated with 1 µM Arteannuin 09 for 48 h. Interestingly, a decrease in the expression of p21 was observed in ATF-4 knocked down conditions compared to alone Arteannuin 09 treated cells (**Figure 4D**). These results collectively suggest that Arteannuin 09 mediated up-regulation of ATF-4 is central in the induction of autophagic influx at early time points (6 h) and senescence at the later time point (48 h).

### 3.5 Arteannuin 09 mediated C/EBPβ and ATF-4 augmentation are essential prerequisite for p21 upregulation to promote cellular senescence

**Figure 5:**
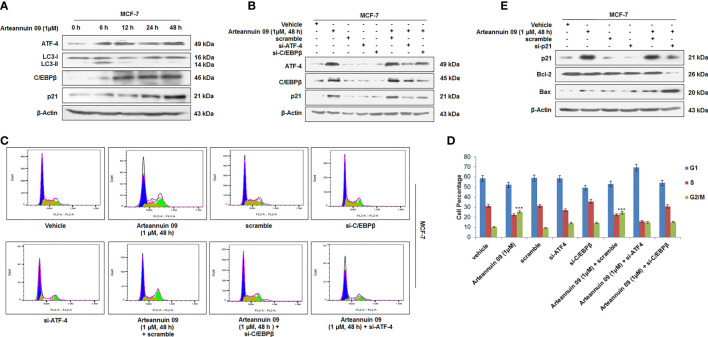
Arteannuin 09 upregulates p21 **(A)** Immunoblotting analysis of the MCF-7 cells after Arteannuin 09 (1 µM) treatment for different time points (0 h to 48 h) for the expression of ATF-4, LC3-I/II, C/EBPβ, and p21. β-actin was taken as the loading control. **(B)** Immunoblot analysis of MCF-7 cells after transient transfections for si-ATF-4 and/or si-C/EBPβ in presence or absence of Arteannuin 09 (1 µM) for 48 h. β-actin was taken as the loading control. **(C, D)** Cell cycle analysis of MCF-7 cells with transiently transfected si-ATF-4 and/or si-C/EBPβ in presence or absence of Arteannuin 09 treatment for 48 h, (N=3). Cell cycle analysis was performed with FlowJo software by employing the Watson (pragmatic) model. Black line represents actual cell population, pink line represents how accurately the model fits the cell population. Blue color represents G_1_, olive S phase and parrot green G_2_/M. **(E)** Immunoblotting analysis of the MCF-7 cells after si-RNA mediated knockdown of p21 and/or treatment of Arteannuin 09 (1 µM) treatment for 48 h. β-actin was taken as the loading control. Densitometry analysis given **Supplementary Information** (section 2.6). Each experiment was carried out in triplicates (N=3) and results (in **Supplementary Section 2.6**) are expressed as mean± sd ***p < 0.001.

While we have established consistent ATF-4 activation by Arteannuin 09 responsible for both autophagy (6 h) and senescence induction (48 h), we were keen to investigate the role of CHOP (GADD153) and C/EBPβ in connection with the decisive shift from autophagy to senescence. Recent reports implicate CHOP (GADD153) and C/EBPβ (belonging to the CCAAT/enhancer-binding protein (C/EBP) family of transcription factors) in inducing growth arrest signals (47–50). Besides its role in apoptosis, several studies have demonstrated the role of C/EBPβ in oncogene-induced senescence (51). Our western blotting analysis did not find any significant change in the expression of CHOP/GADD153 (**Supplementary Figures 2, 3A**). Interestingly, at 6 h time point Arteannuin 09 induced ATF-4 but not C/EBPβ, suggesting that ATF-4 is an important mediator of autophagic response of Arteannuin 09 (**Figure 5A**). Nevertheless, at higher time points (12 to 48 h), protein expression of C/EBPβ was eminent, in concomitance with the upregulation of p21. To examine whether C/EBPβ has any role in mediating the senescent response of Arteannuin 09, we transfected MCF-7 cells with si-C/EBPβ/si-ATF-4 followed by Arteannuin 09 treatment. Immuno-blotting analysis of MCF-7 cells showed a robust decrease in p21 in si-ATF-4 plus Arteannuin 09 and si-C/EBPβ plus Arteannuin 09 treated cells compared to alone Arteannuin 09 treated conditions, implying the decisive shift from autophagy to senescence requires constant ATF-4 activation and time dependent C/EBPβ upregulation (**Figure 5B**). Again to validate the above results, we performed cell cycle analysis of MCF-7 cells with si-RNA mediated knockdown of ATF-4 or C/EBPβ, followed by Arteannuin 09 treatment for 48 h (**Figures 5C, D**). The results also mimicked our immuno-blotting results that both ATF-4 and C/EBPβ are required for G_2_/M cell cycle arrest mediated by p21. Furthermore, SAHF assay was performed with the same experimental setup as above in MCF-7 cells. Beaded nuclei were observed only in Arteannuin 09 treated MCF-7 cells. Contrastingly, in cells where either C/EBPβ or ATF-4 was knocked down, Arteannuin 09 failed to induce the beaded nuclear morphology (**Supplementary Figures 2, 3B**). To further characterize the role of ATF-4 and C/EBPβ mediated upregulation of p21 in cell cycle control during ER stress, we knocked down p21 by siRNA in MCF-7 cells following the Arteannuin 09 treatment (**Figure 5E**). As shown in **Figure 5E** si-RNA mediated knock down of p21 and Arteannuin 09 treatment resulted in the upregulation of apoptotic marker, viz. Bax and downregulation of survival marker Bcl-2, suggesting the pro-senescence and antiproliferative role of p21. Therefore, all these above results suggested that the ATF-4 and C/EBPβ induced p21 upregulation promotes cellular senescence in Arteannuin 09 treatment at 48 h.

### 3.6 Arteannuin 09 augments the ATF-4 and C/EBPβ physical interaction

**Figure 6:**
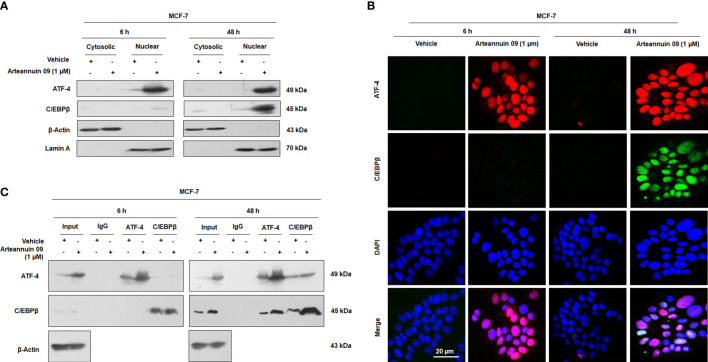
Arteannuin 09 promotes interaction of ATF-4 and C/EBPβ. **(A)** Western blot analysis from fractionation (cytosolic and nuclear) studies to ascertain the localization of ATF-4 and C/EBPβ in presence of Arteannuin 09 treatment in MCF-7 cells. β-Actin was used as a cytosolic loading control and Lamin A as a nuclear loading control (N=3). **(B)** Co-immunocytochemistry of ATF-4 and C/EBPβ at 6 h and 48 h. N=3. Magnification 20X, scale bar–20 µm. **(C)** Co-immunoprecipitation (co-IP) assay showing interaction of ATF-4 and C/EBPβ in Arteannuin 09 treated MCF-7 cells (N=3).

ATF-4 like other bZIP proteins must dimerize to bind and switch on genes (19, 52–54). Nonetheless, homodimers of ATF-4 are unstable (20). C/EBPβ, a bZIP protein itself, has been reported as a binding partner of ATF-4 to form ATF-4/C/EBPβ functional heterodimer complexes (20, 54). As seen in the previous results, there was a significant increase in the expression of C/EBPβ at 48 h. These deliberations led us to hypothesize that ATF-4 and C/EBPβ may physically interact with each other to form transcriptionally active heterodimer complexes. To test this hypothesis, we performed fractionation, co-localization and co-immunoprecipitation studies in MCF-7 cells. Interestingly, our fractionation studies revealed the nuclear expression of ATF-4 at both 6 h and 48 h, while as C/EBPβ expression was only observed in nuclear fractions at 48 h (**Figure 6A**). Similarly, dual immunocytochemistry of ATF-4 and C/EBPβ unveiled a nuclear co-localization pattern upon Arteannuin 09 treatment at 48 h with no such pattern being observed at 6 h (**Figure 6B**). Additionally, co-immunoprecipitation results demonstrated that Arteannuin 09 treatment at 48 h leads to the enrichment of ATF-4/C/EBPβ interaction as evidenced from enhanced pull down of ATF-4 by C/EBPβ and C/EBPβ pull down of ATF-4 (**Figure 6C**). Strikingly, no such assortment of ATF-4/C/EBPβ interaction was observed in Arteannuin 09 treated MCF-7 cells at 6 h. These results collectively suggest that Arteannuin 09 triggers the physical binding of ATF-4 and C/EBPβ, potentially resulting in ATF-4/C/EBPβ hetero-dimerization to maintain homeostasis by halting cell progression *via* G_2_/M.

### 3.7 Inhibition of autophagy conferred senescence to apoptosis switching in presence of Arteannuin 09

**Figure 7:**
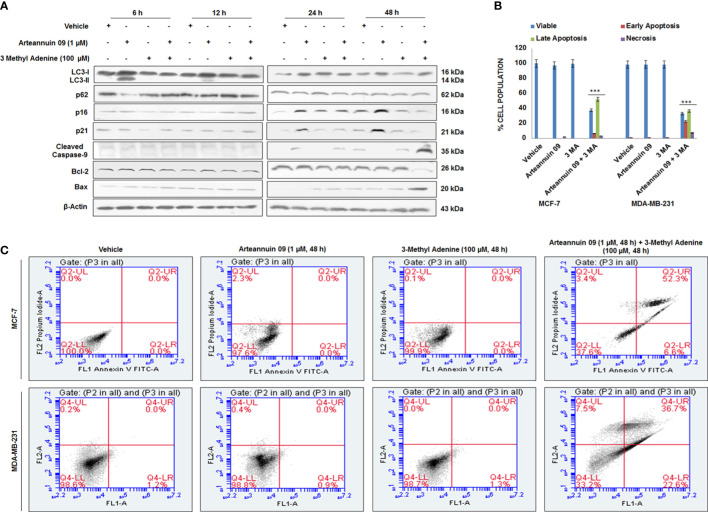
Arteannuin 09 switches the fate of cells from senescence to apoptosis in presence of 3-Methyl Adenine **(A)** Immunoblotting analysis of MCF-7 for the expression of autophagy markers LC3-I/II & p62, senescence markers p16 & p21, and apoptotic markers Bax & Cleaved Caspase 9 after treatment of vehicle, Arteannuin 09 (1 µM) in presence or absence of 3 Methyl Adenine (100 µM) for the indicated time points. β-actin was taken as the loading control. **(B, C)** Annexin V-FITC analysis of MCF-7 cells, following the Arteannuin 09 (1 µM) treatment in presence or absence of 3 Methyl Adenine (100 µM) for 48 h (N=3). Densitometry analysis given in **Supplementary Information** (section 2.6). Each experiment was carried out in triplicates (N=3) and results are expressed as mean± sd ***p < 0.001.

In drug-induced protective autophagy, autophagy inhibition often leads to an enhanced apoptotic response (55). We assumed that if, autophagy is a resistance mechanism to Arteannuin 09 induced apoptosis, we should witness apoptotic induction following autophagy inhibition in presence of Arteannuin 09. To test this hypothesis, we used 3-methyladenine (3-MA) as an inhibitor of autophagy. 3-MA, a class III PI3K inhibitor, is extensively used to block autophagic activation (56). Therefore to check the fate of cells in Arteannuin 09 treated as well as autophagy blocking conditions, we performed the western blotting analysis of all significant autophagy markers (LC3-I/II, p62), senescence markers (p16, p21), and apoptotic markers (Bax, Cleaved Caspase 9) from 6 h to 48 h. Our results uncovered a consistent amplification in the apoptotic markers (Bax and cleaved caspase 9) and a decrease in survival marker Bcl-2 at 48 hours in Arteannuin 09 plus 3-MA co-treated cells (**Figure 7A**). However, we also conducted Annexin V and propidium iodide staining to confirm these results. The results unveiled that most MCF-7 and MDA-MB-231 cells at 48 h entered the apoptotic quadrants when co-treated with Arteannuin 09 and 3-MA (**Figures 7B, C**). Moreover, as post transient knockdown of p21 and treatment with Arteannuin 09 conferred apoptotic capability to MCF-7 cells (**Figure 5C**). We sought to investigate the role of p53, one of the key protein involved in intrinsic apoptotic pathway. We treated MCF-7 cells with Arteannuin 09 after transient knockdown with si-p21. We did not observe any significant change in the expression of p53 in transiently knocked down p21 condition (**Supplementary Figure 2.4A**). Same results were seen in the Arteannuin 09 and 3-MA cotreated MCF-7 cells (**Supplementary Figures 2, 4B**). Collectively, these results demonstrated that inhibition of autophagy by 3-MA shifted the Arteannuin 09 response from senescence to apoptosis. Therefore, Arteannuin 09 mediated autophagy has a pro survival role.

### 3.8 Arteannuin 09 is an effective inhibitor of tumor growth *in vivo*

**Figure 8:**
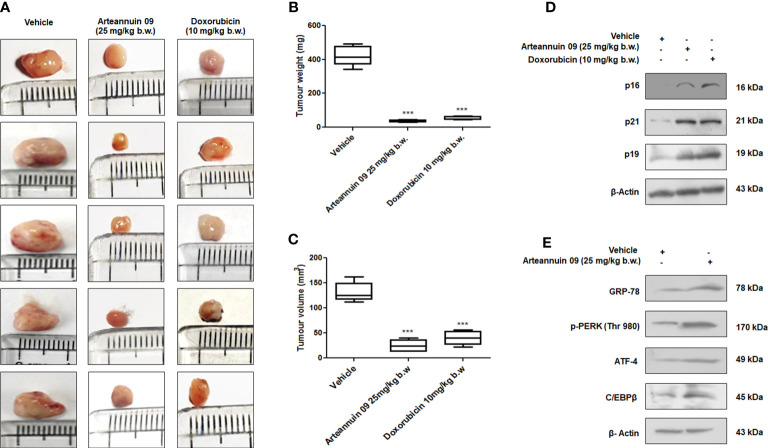
Arteannuin 09 impedes *in vivo* tumor growth **(A)** Images representing the tumor growth in vehicle, Arteannuin 09 (25 mg/kg b.w.) and doxorubicin (10 mg/kg b.w.) treated 4T1 mouse mammary carcinoma model. **(B, C)** Quantification of tumor volume and weight from the above groups of animals respectively, n=5. **(D)** Immunoblotting analysis of p16, p21 and p19 in tumor samples collected from vehicle, Arteannuin 09, and doxorubicin treated groups of animals. **(E)** Immunoblotting analysis of GRP-78, p-PERK, ATF-4, and C/EBPβ in tumor samples collected from vehicle and Arteannuin 09 treated groups of animals. Densitometry analysis given in **Supplementary Information** (section 2.6). Each experiment was carried out in triplicates (N=3) and results are expressed as mean± sd ***p < 0.001.

As the onset of premature senescence obstructs the growth of the primary tumor and further cancer progression, to evaluate the *in vivo* efficacy of Arteannuin 09, we employed the 4T1 mouse mammary carcinoma model. Intraperitoneal administration of Arteannuin 09 **(**25 mg/kg b.w.) was performed in each animal on alternate days for two weeks. The results depicted a 75% inhibition in tumor volume compared to the vehicle-treated group (**Figures 8A, B**). There was a significant reduction in tumor weight as our results demonstrated a 63% reduction in Arteannuin 09 treated groups compared to vehicle-treated groups (**Figure 8C**). Similarly, western blot analysis of the tumor samples mimicked our *in vitro* results (**Figures 8D, E**). The protein expression of p16, p19, p21 as well as GRP-78, p-PERK, ATF-4 and C/EBPβ was elevated in Arteannuin 09 treated cohort. Therefore, these results firmly imply that Arteannuin 09 is a potent inhibitor of tumor growth, inducer of ER stress and premature senescence *in vivo*.

## 4 Discussion

Acquired by organisms from unicellular to the multicellular level organization, protective autophagy, a cellular adaptation, helps avoid stressful conditions (57). Based on the severity and type of the stress, cells trigger autophagy, cell cycle arrest, or apoptosis underlying the prevalent cellular signaling cascade. Cancer cells explore the various survival mechanisms to cope with environmental stresses and become addictive to these pathways. In this study, we demonstrate that in presence of Arteannuin 09, there was consistent ER stress mediated regulation of p21 expression by ATF-4 and that p21 is a pro-survival effector of ATF-4. Many recent evidences have convincingly shown the dependency of p21 expression by diverse mechanisms falling under ER stress. Even though numerous positive reinforcement mechanisms exist within the UPR, our work identifies novel cross-regulation between two key UPR branches; autophagy and cell cycle arrest. By attenuating autophagy, ATF-4 and CEBP/β seem to reinforce adaptive efforts mediated through pAMPK, LC3I/II, p62, and Beclin-1; moreover, it removes a critical apoptotic hurdle mounted by Bcl-2/Bax mediated intrinsic pathway shift into cell cycle arrest.

In the pursuit of anti-cancer drug development, targeting UPR components may bear fruitful results as tumor cells are prone to physiological stresses such as nutrient deprivation, acidosis, hypoxia etc., and therefore rely on UPR components for survival (58, 59). Hence, targeting UPR components to reprogram signaling pathways so as to shift the balance towards pro-apoptotic arm rather than pro-adaptation could provide vital breakthrough in the emerging area of diagnosis. However, mild ER stress serves as an adaptive, pro-survival response which resolves the stress associated with the accumulation of unfolded or misfolded proteins and reinstates homeostasis. Contrast, prolonged ER stress often tend to trigger cell death and in this context it is well known that in response to ER stress, ATF-4 induction induces apoptosis (60, 61). Intriguingly, we found a consistent protective autophagy cascade in the initial hours (0-6 h) of Arteannuin 09 treatment through inhibition of mTOR downstream target p-S6K and increased expression of autophagy protein markers ATG5, Beclin-1, and ATG7; however, there was strong occurrence of emerging senescence morphology at later time points (48h onwards) by ATF-4 mediated phosphorylation of AMPK at Thr172. Our findings suggested the mechanism by which Arteannuin 09 activated AMPK pathway could be mediated by ATF-4 activation.

The involvement of p21 in G_2_/M regulation and the association of prolonged G_2_ arrest with senescence phenotype are emerging field of research. Although G_2_/M arrest are often intricately converged into promoting apoptosis but recent evidences suggest a continuous G_2_/M arrest lead to activate premature senescence. Autophagy can augment the pro- and anti-tumorigenic effects depending on the cellular context, while also exerting an essential immune modulatory function. Autophagy is frequently accompanied by senescence, at least in major clinically relevant doses of drugs (62). On the other hand, senescence has been deeply corroborated into an irreversible form of growth arrest. Although, both autophagy and senescence frequently occur in parallel in reaction to therapeutic exposure and having common key regulators, such as p53 and mammalian target of rapamycin (mTOR), whether they are self-regulating or interdependent is still not clear (62). In this research we showed a distinct switching of Autophagy to senescence by modulating mTOR downstream target p-S6K at the prolong exposure of MCF-7 and MDA-MB-231 cells to Arteannuin 09. The results not only uncovered a sharp increase in the conventional G_2_/M arrest markers, *viz*. Cyclin b and Cdk-1, also significantly augmented the expression of senescence hallmarks (p21, p16, and p19; β galactosidase activity and senescence-associated heterochromatin foci) from 48 h to 72 h of Arteannuin 09 treatment.

**Figure 9:**
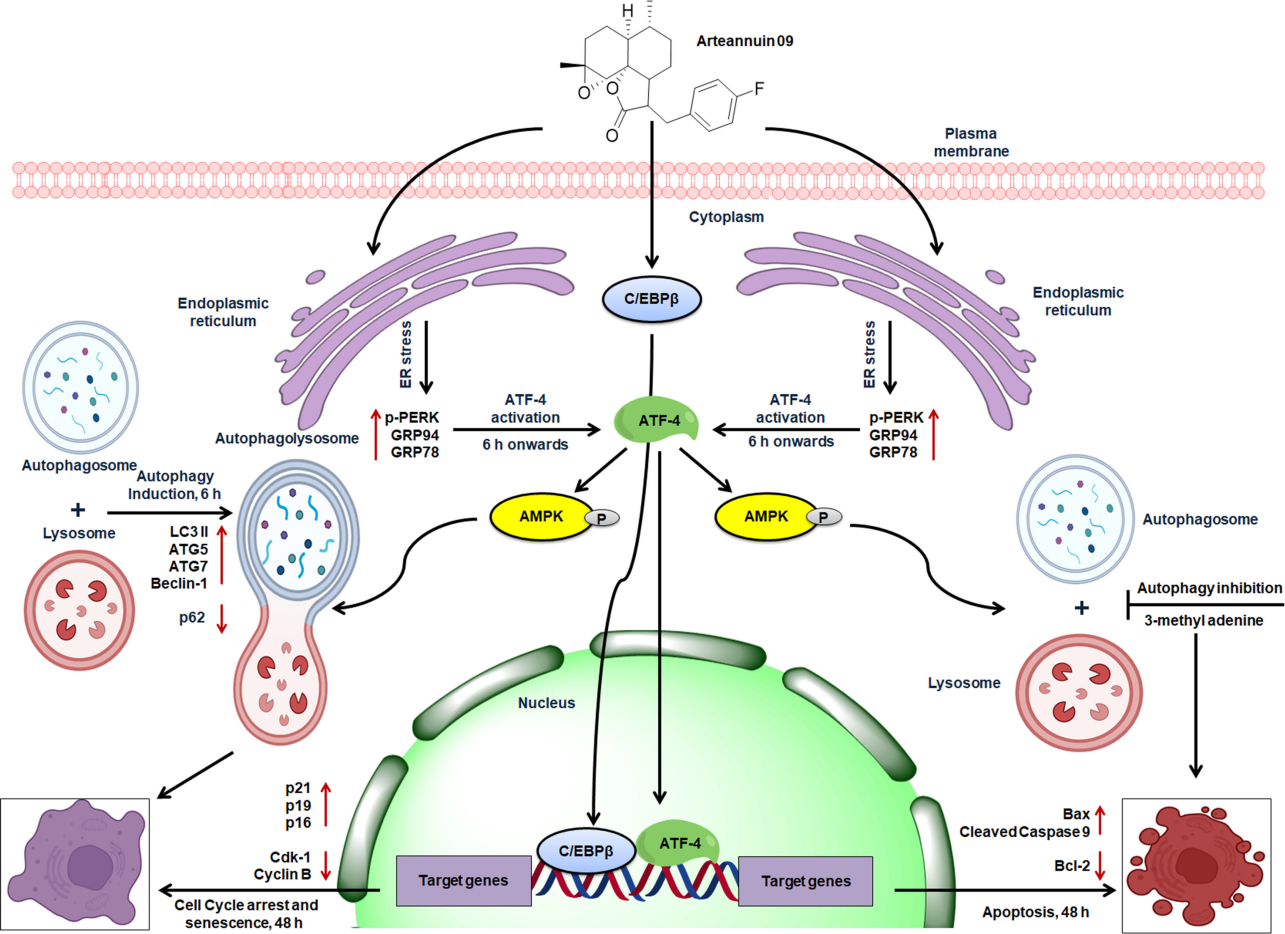
Schematic representation of the proposed mechanism of action of Arteannuin 09 in breast cancer cell lines, MCF-7 and MDA-MB-231. Arteannuin 09 promotes the Autophagy to senescence switching regulated by ER stress response.

We also demonstrated that the regulation of p21 expression is dependent on both ATF-4 and C/EBPβ. ATF-4 homodimers are unstable (20), consequently, ATF-4 like other bZIP proteins must dimerize to bind and switch on genes (19, 52–54). Several studies have shown that ATF-4 bZIP domain has least affinity with another ATF-4 bZIP domain compared to its affinity for the bZIP domains of several other bZIP family members (63, 64). Our co-localization and fractionation studies revealed nuclear expression of both ATF-4 and C/EBPβ at 48 h. while as immuno-precipitation studies evidenced enhanced pull down of ATF-4 by C/EBPβ and C/EBPβ pull down of ATF-4. Considering the above results coupled with previous findings, Arteannuin 09 might trigger the physical binding of ATF-4 and C/EBPβ, potentially resulting in ATF-4/C/EBPβ hetero-dimerization to maintain homeostasis by halting cell progression *via* G_2_/M (**Figure 9**).

García et al. (65) suggests autophagy imperative for maintaining stemness by preventing senescence in muscle cells and at the same time autophagy is also reported to be essential for senescence (10, 66). Our results can reconcile these conflicting findings. Autophagy might prevent senescence by acting as a frontline stress reliever. However, if stress is persistent and also to an extent that is not enough for apoptosis but can damage cellular dynamics/integrity, cells may prefer senescence i.e., a stable cell cycle arrest. Alternatively, obstructing “protective autophagy” can be another strategy to hinder tumor growth under ER stress conditions. In a study, thapsigargin induced ER stress in MDA-MB-231 cells led to the accumulation of ATF-4, SESN2, and DDIT4 at protein and mRNA levels, decrease in the phosphorylation of mTORC1 substrates including p70S6K and 4EBP1 was observed as well, suggesting that mTORC1 inhibition may be a general cellular event following ER stress (15). However, we have found a distinct correlation of ER stress mediated ATF-4 upregulation with AMPK activation which is an essential prerequisite for autophagy induction leading to an ultimate senescence cascade in breast cancer cells. AMPK signaling pathway regulates the energy balance in eukaryotes. Emerging evidences from recent studies suggest the direct correlation of UPR and AMPK in the pathogenesis of many diseases including cancer (67). ATF-4 is also responsible for regulating stress responses in mammalian cells. Additionally, the role of AMPK in the early activation of UPR and related genes such as ATF-4 is consistently emerging (68).

Autophagy works as a quick response team to restore the homeostasis in retaliation to the received threat and thus salvage components/nutrients to avoid cell death. Notably, in most of the current literature, autophagy is implied as a cytoprotective function; and inhibition of cytoprotective autophagy would be anticipated to reboot chemosensitization (69, 70). Here we demonstrated that inhibition of autophagy by 3-MA shifted the Arteannuin 09 response triggering apoptosis by amplification in the apoptotic markers (Bax and cleaved caspase 9) and a decrease in survival marker Bcl-2 at 48 hours in Arteannuin 09 plus 3-MA co-treated cells and thus avoiding senescence.

The only clinically approved drug for autophagy inhibition is Hydroxychloroquine (71). However, even higher doses of the drug *in vivo* produce modest autophagy inhibition. Additionally, in acidic environments, it fails to inhibit autophagy because of reduced cellular uptake of the drug (72, 73). Therefore, there is a need for a combinatorial approaches rather than monotherapy to minimize the toxicity issues caused by high doses and at the same time increase the efficiency. In our study, we co-treated MCF-7 cells with Arteannuin 09 and autophagy inhibitor 3-methyl adenine which drastically reduced survival protein Bcl-2 and increased the apoptotic proteins. Hence, to improve the autophagy-related cancer therapy, there is a requirement to understand better the dual faced role of autophagy in tumor survival, the recognition of tumor sub-populations that will be prone to autophagy inhibition and better apprehension of interplay between tumor cells and immune system.

## 5 Conclusion

We have explored the potential of Arteannuin 09 for the induction of autophagy, established the molecular mechanism of autophagy induction by Arteannuin 09; and demonstrated that Arteannuin 09 *via* “protective autophagy” can lead to cell cycle arrest/senescence in a manner that may be an escape response to apoptosis in breast cancer cells. This work will help in understanding the molecular mechanism of senescence induction *via* ER stress by Arteannuin 09 and other pharmacologically relevant scaffolds. Our results suggest that ATF-4 and C/EBPβ mediated p21 induction plays a vital role in the cellular response to endoplasmic stress and induction of p21, a well-known cell cycle inhibitor, is likely in response to ER stress as a protective take to safeguard the genomic integrity. The data presented in this study, will strengthen the idea of targeting protective autophagy as an alternative therapeutic approach against cancer.

## Data availability statement

The original contributions presented in the study are included in the article/**Supplementary Material**. Further inquiries can be directed to the corresponding author.

## Ethics statement

The animal study was reviewed and approved by “Committee for the Purpose of Control and Supervision of Experiments on Animals” (CPCSEA, New Delhi, India) and Institutional animal ethics committee (IAEC No: 266/79/8/2021).

## Author contributions

AG: Supervision, Conceptualization, and funding acquisition. KM: Conceptualization, Methodology, Writing- Original draft preparation, *in vitro* and *in vivo* experiments. MF: Writing - Review & Editing and Graphical abstract. SA: Data analysis and Densitometry. TA: Immunocytochemistry and western blotting experiments. JR: *β*-(4-fluorobenzyl) Arteannuin B (Arteannuin 09) synthesis. SC: β-gal assay. AA: *β*-(4-fluorobenzyl) Arteannuin B (Arteannuin 09) synthesis. MB: Facilitated FACS experiments. ZA: Data analysis. All authors contributed to the article and approved the submitted version.

## Acknowledgments

The work was supported by the internal institutional grant (MLP-6002) from the Council of Scientific and Industrial Research (CSIR), Government of India. The authors acknowledge the funding agency Council of Scientific and Industrial Research (CSIR) and University Grants Commission (UGC) for providing fellowships to the research scholars. We also thank our worthy director Dr. D. Srinivasa Reddy for his encouragement to complete this work. This manuscript has been assigned the institutional publication number CSIR-IIIM/IPR/00428.

## Conflict of interest

The authors declare that the research was conducted in the absence of any commercial or financial relationships that could be construed as a potential conflict of interest.

## Publisher’s note

All claims expressed in this article are solely those of the authors and do not necessarily represent those of their affiliated organizations, or those of the publisher, the editors and the reviewers. Any product that may be evaluated in this article, or claim that may be made by its manufacturer, is not guaranteed or endorsed by the publisher.
