# Supplementary material for: β-(4-fluorobenzyl) Arteannuin B induced interaction of ATF-4 and C/EBPβ mediates the transition of breast cancer cells from autophagy to senescence
Source: Front Oncol. 2022 Nov 17;12:1013500. doi: 10.3389/fonc.2022.1013500 (PMC9713483; doi:10.3389/fonc.2022.1013500)
Supplement: Supplementary file 1 [file DataSheet_1.docx]

**Supplementary Information**

***β*-(4-fluorobenzyl) Arteannuin B induced interaction of ATF-4 and C/EBPβ mediates the transition of breast cancer cells from autophagy to senescence**

Khalid Bashir Mir ^1,2^ , Mir Mohd Faheem ^2,3^, Syed Mudabir Ahmad ^1,2†^ , Javeed Ur Rasool ^1,4†^, Tanzeeba Amin ^1,2†^, Souneek Chakraborty ^1^, Madhulika Bhagat ^3^, Zabeer Ahmed ^1,2^, Asif Ali ^1,4,5*^, Anindya Goswami ^1,2*^

1*. Academy of Scientific & Innovative Research (AcSIR), Ghaziabad 201002, India*

2*. Pharmacology Division, CSIR-Indian Indian Institute of Integrative Medicine, Jammu 180001, India*

3*. School of Biotechnology, University of Jammu, Jammu, 180006, India*

4*. Natural Product and Medicinal Chemistry Division, CSIR-Indian Institute of Integrative Medicine, Canal Road, Jammu 180001, India*

5*. Division Of Medicinal and Process chemistry,* *CSIR-Central Drug Research Institute, Sector 10, Jankipuram Extension, Sitapur Road, Lucknow, Uttar Pradesh, 226031, India*

† *Contributed equally*

*** Corresponding Author:**

1. Asif Ali, PhD

CSIR-Central Drug Research Institute

Sector 10, Jankipuram Extension, Sitapur Road,

Lucknow, Uttar Pradesh, 226031, India

E-mail: [asifchem73@gmail.com](mailto:asifchem73@gmail.com)

1. Anindya Goswami, PhD

CSIR-Indian Institute of Integrative Medicine,

Canal Road, Jammu 180001, India

Tel.: +91 019102569111: fax: +91 01912569333

E-mail: [agoswami@iiim.ac.in](mailto:agoswami@iiim.ac.in); [agoswami@iiim.res.in](mailto:agoswami@iiim.res.in)

1. **Chemistry**
   1. **Synthesis**

The compound Arteannuin 09 (3i) was synthesized as described in our previous publication. (<https://doi.org/10.1016/j.bioorg.2022.105694>)

- 1. **Structure of Arteannuin 09 (3i)**

- 1. **NMR**

**1H and 13C, DEPT NMR Spectra 3i**

- 1. **HPLC**

**HPLC purity data of 3i.** (unwanted values in the table are not shown for clarity)


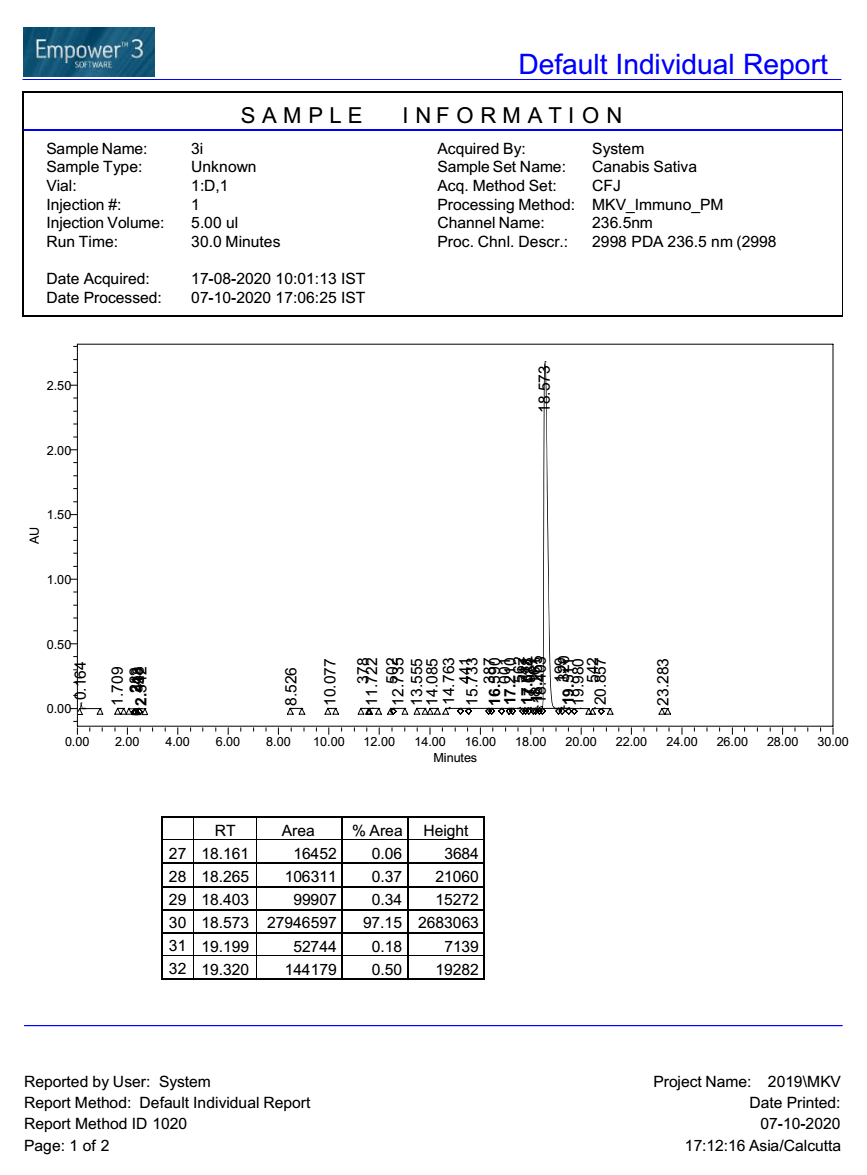


**2. Results**

**2.1. Autophagy screening for Arteannuin derivatives**


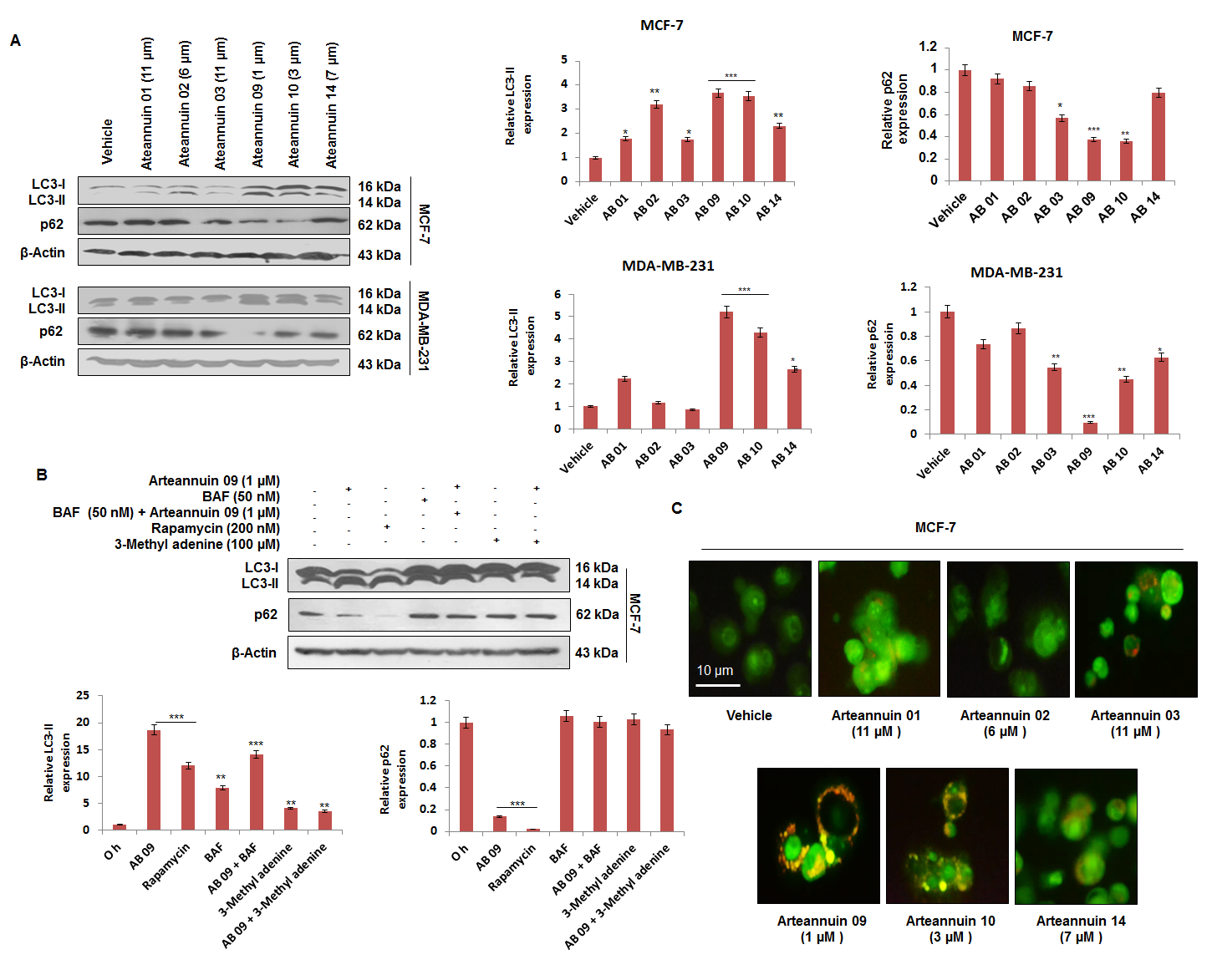


**Supplementary Figure 2.1:**  Screening of Arteannuin B derivatives for Autophagic flux. (A) MCF-7 and MDA-MB-231 cells were treated with different concentrations of most active (based on IC_50_) Arteannuin B derivatives. Western blotting of LC3I/II and p62 proteins in MCF-7 and MDA-MB-231 cell lines. (B) Western blotting of LC3I/II and p62 proteins in MCF-7. (C) Acridine orange staining for autophagy induction. Magnification 20X and scale bar – 10 µm. Each experiment was carried out in triplicates (N=3) and results are expressed as mean± sd ****p < 0.001, **p < 0.01, *p <0.05.*

**2.2. Immunocytochemistry of MDA-MB-231 cells**


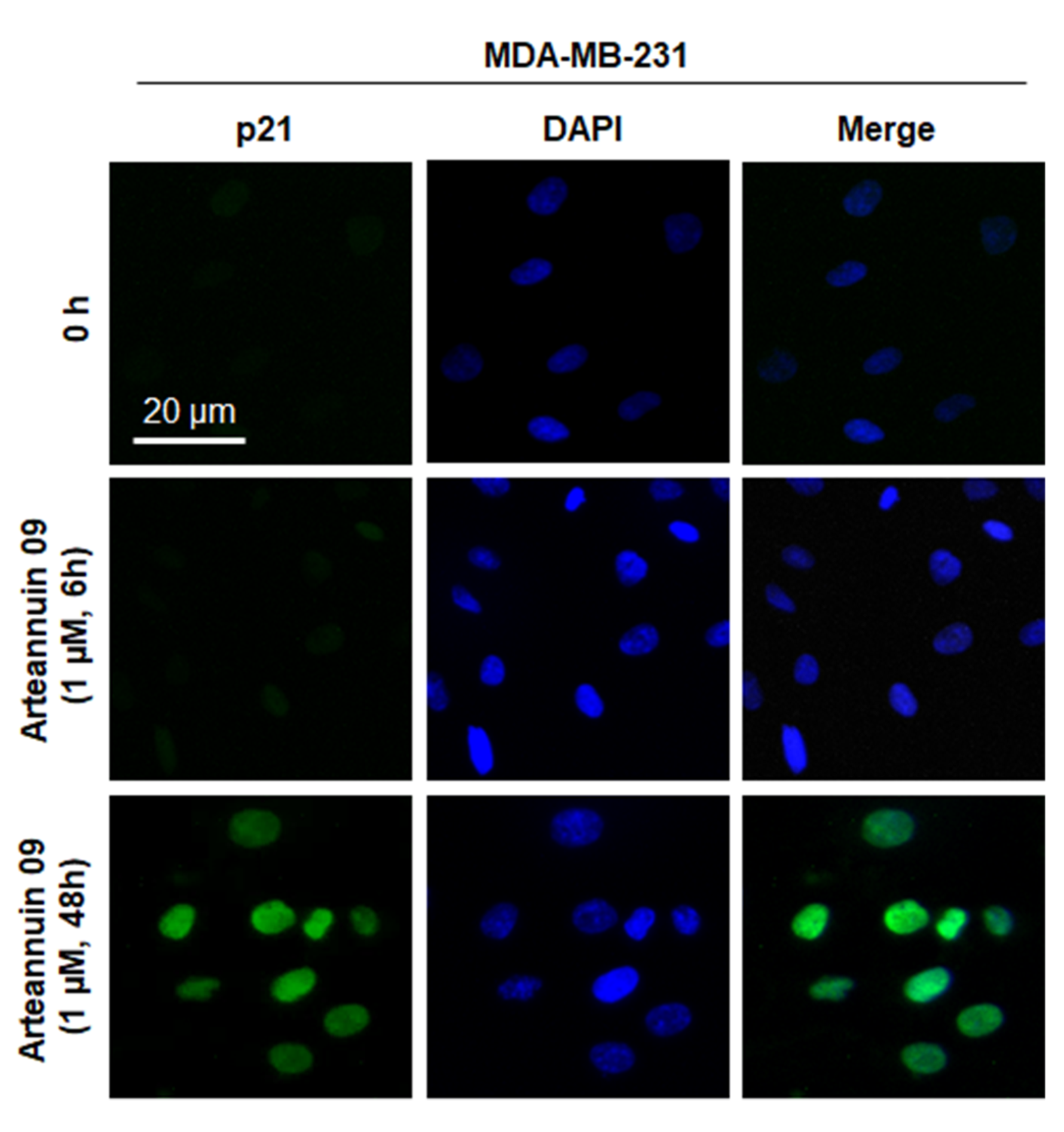


**Supplementary Figure 2.2:** p21 expression in MDA-MB-231 cells. Arteannuin 09 (1 µM, 48 h) treated MDA-MB-231 cells showing nuclear p21 expression. Images were captured under Floid Cell Imaging Station at 20X magnification having a scale bar – 20 µm, The data represents three independent experiments performed separately.

**2.3: CHOP expression and SAHF assay**

**
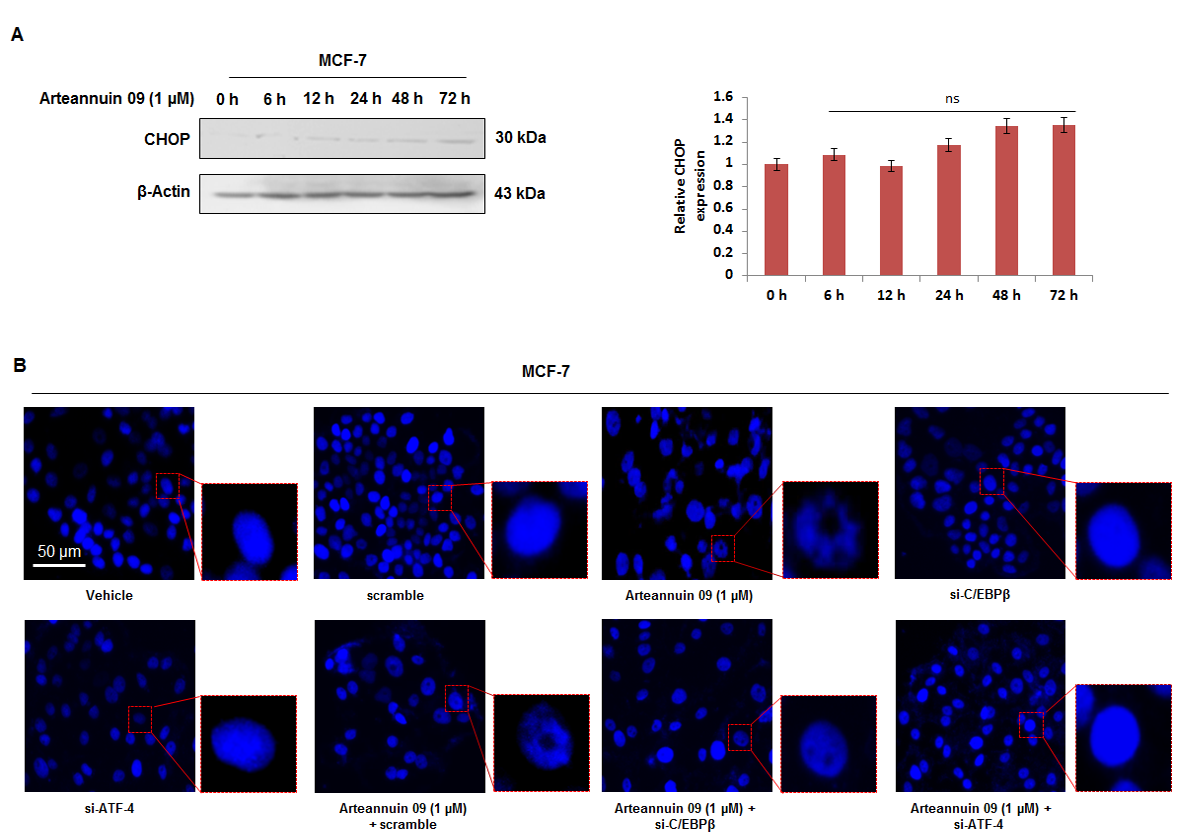
**

**Supplementary Figure 2.3: (A-B)** Immunoblotting of CHOP/GAD153 and its densitometric analysis. β-Actin was taken as loading control**. (C)** SAHF analysis of MCF-7 cells after transient transfections for si-ATF-4 and/or si-C/EBPβ in presence or absence of Arteannuin 09 (1 µM, 48 h). Magnification 20X and scale bar – 50 µm. Each experiment was carried out in triplicates (N=3) and results are expressed as mean± sd, *ns=non-significant.*


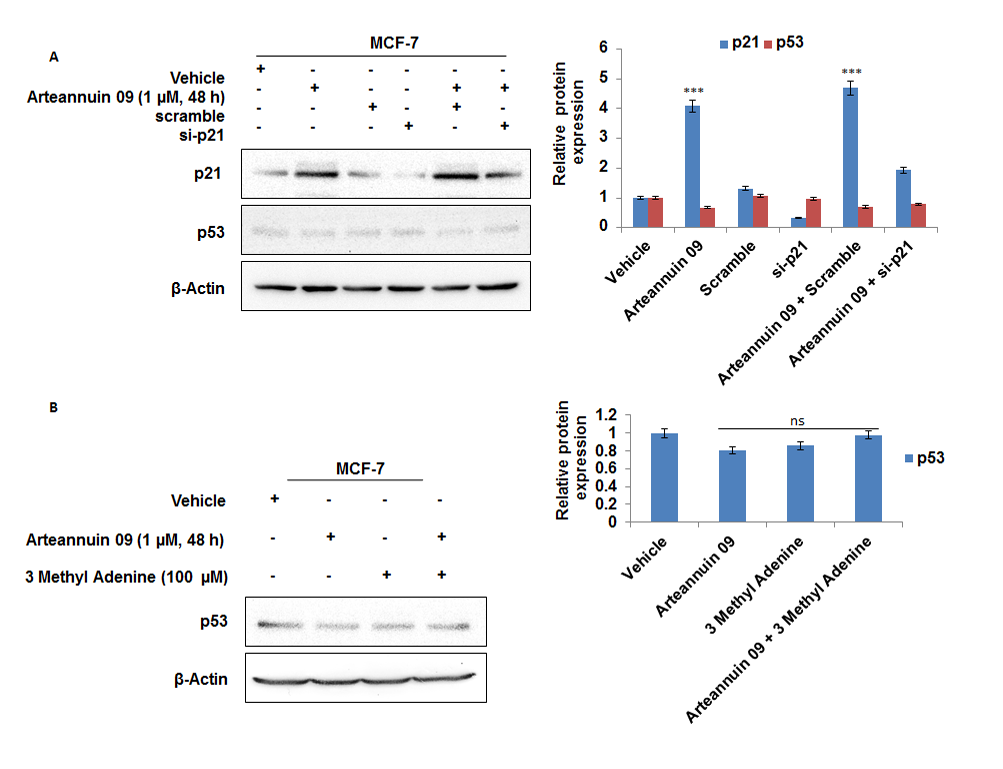
**2.4: p53 expression under si-p21 and 3 Methyl Adenine co-treated condition.**

**Supplementary Figure 2.4: (A-B)** Immunoblotting of p53 and its densitometric analysis. β-Actin was taken as loading control. Each experiment was carried out in triplicates (N=3) and results are expressed as mean± sd ****p < 0.001, **p < 0.01, *p <0.05., ns=non-significant.*

**2.5: Dose response study**

Dose-response study of Arteannuin 09 in Balb/c mice. Arteannuin 09 was administered into the normal Balb/c mice (without tumor) at increasing doses, i.e. 5, 10, 25, 50, 100 and 200 mg/kg b.w. to six separate group of animals. The doses were given intraperitoneally on each alternate day for ten days and animals were observed for any deleterious effects. Dose of 25 mg/kg, b.w. of Arteannuin 09 was selected for treatment to the tumor bearing animals.

| **Arteannuin 09 dosage (ip)** | 5 mg/kg b.w | 10 mg/kg b.w | 25 mg/kg b.w | 50 mg/kg b.w | 100 mg/kg b.w | 200 mg/kg b.w |
| --- | --- | --- | --- | --- | --- | --- |
| **No. of animals** | 5 | 5 | 5 | 5 | 5 | 5 |
| **Mortality** | 0 | 0 | 0 | 1 | 3 | 4 |


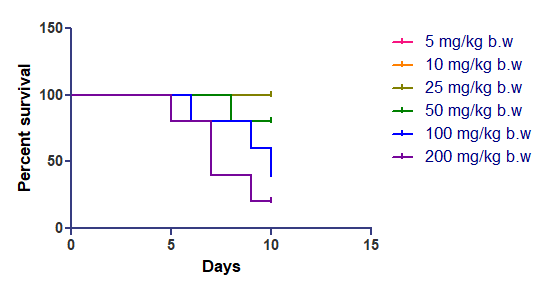


**kaplan meier survival curve**

- 1. **Densitometry analysis**

**FIGURE 1**


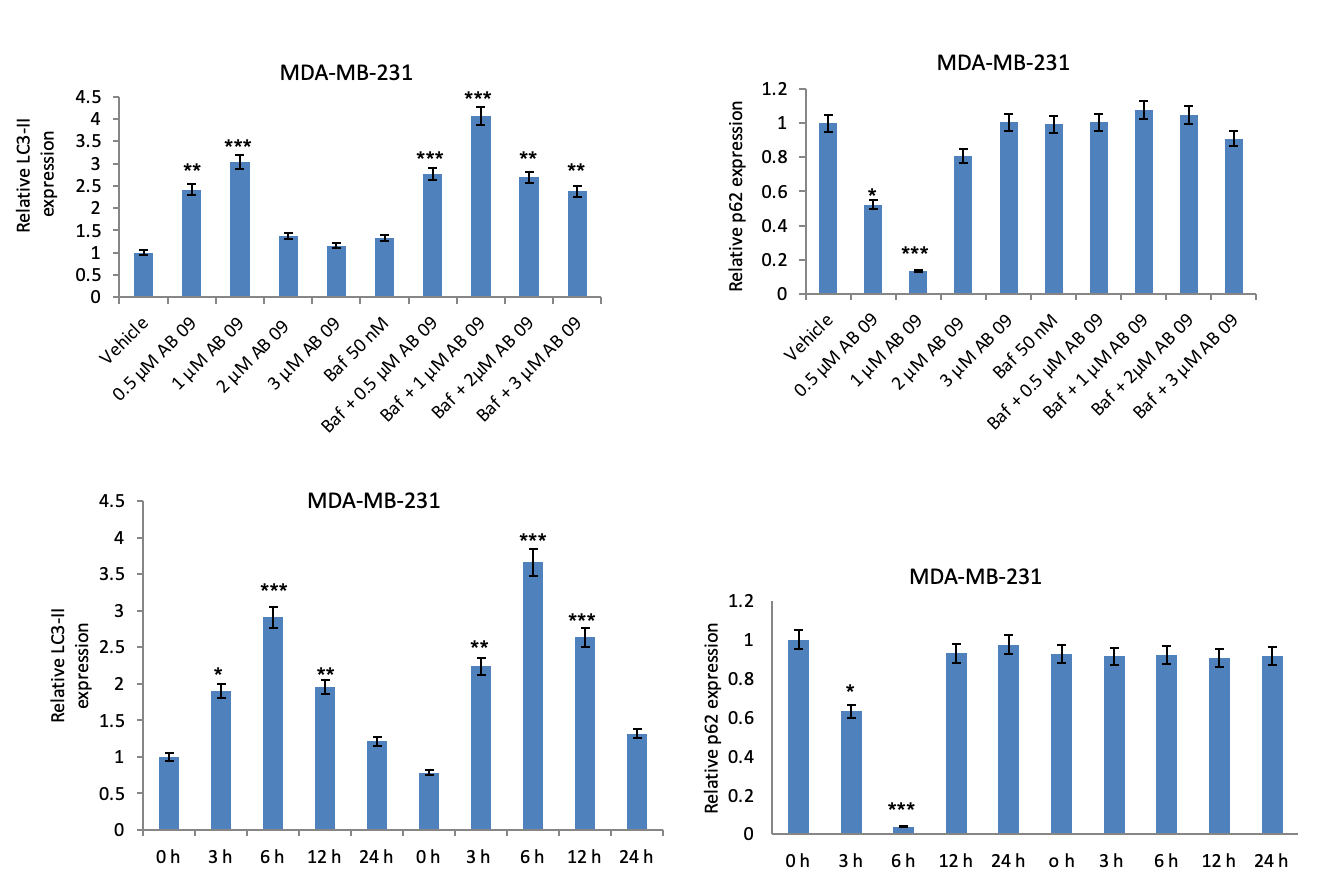


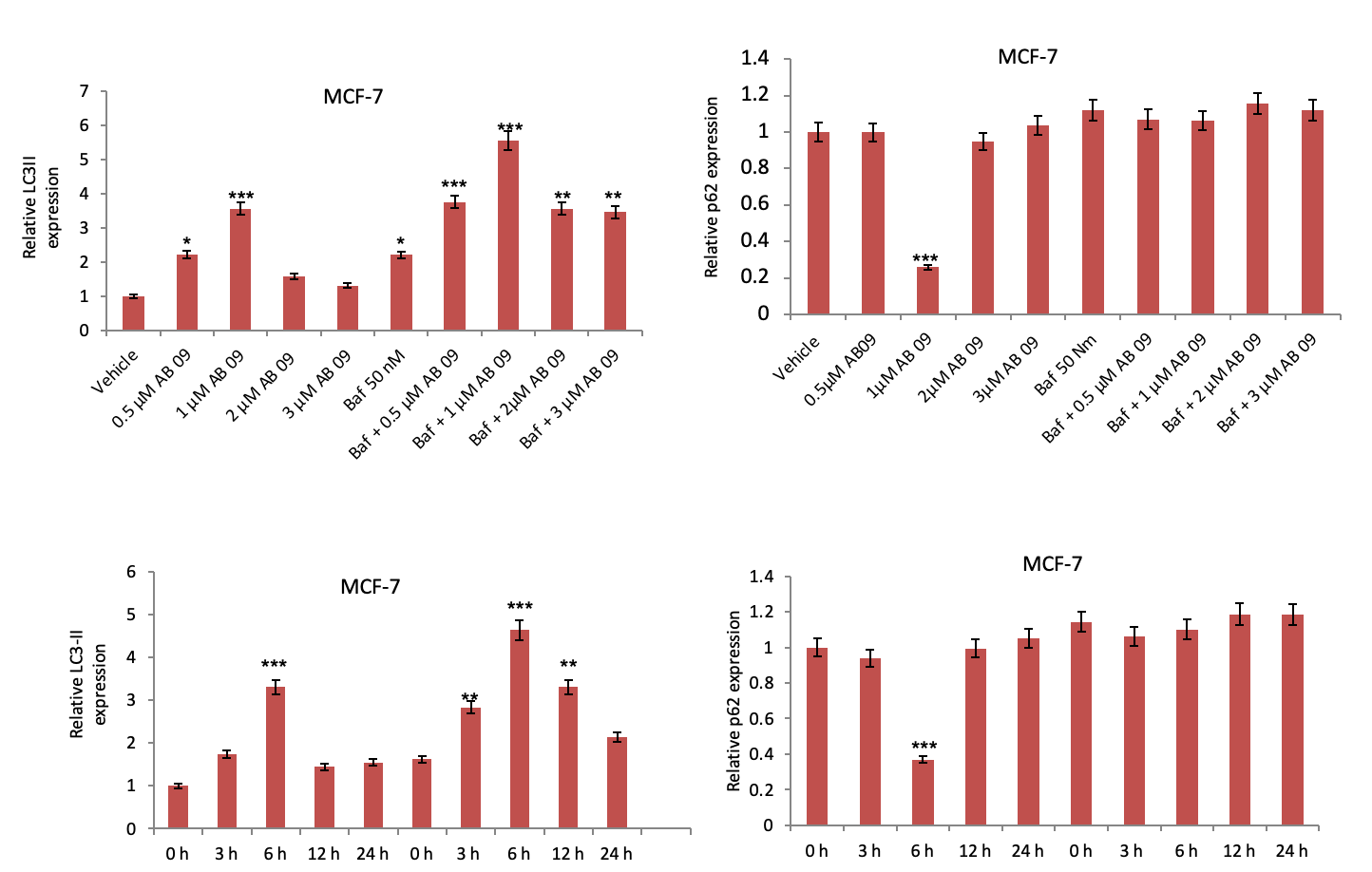


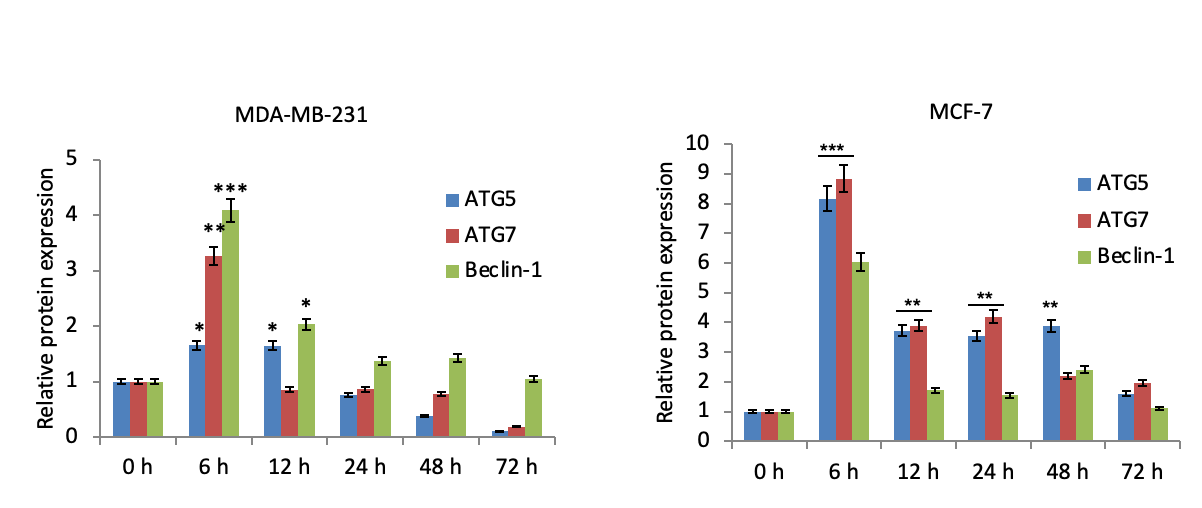


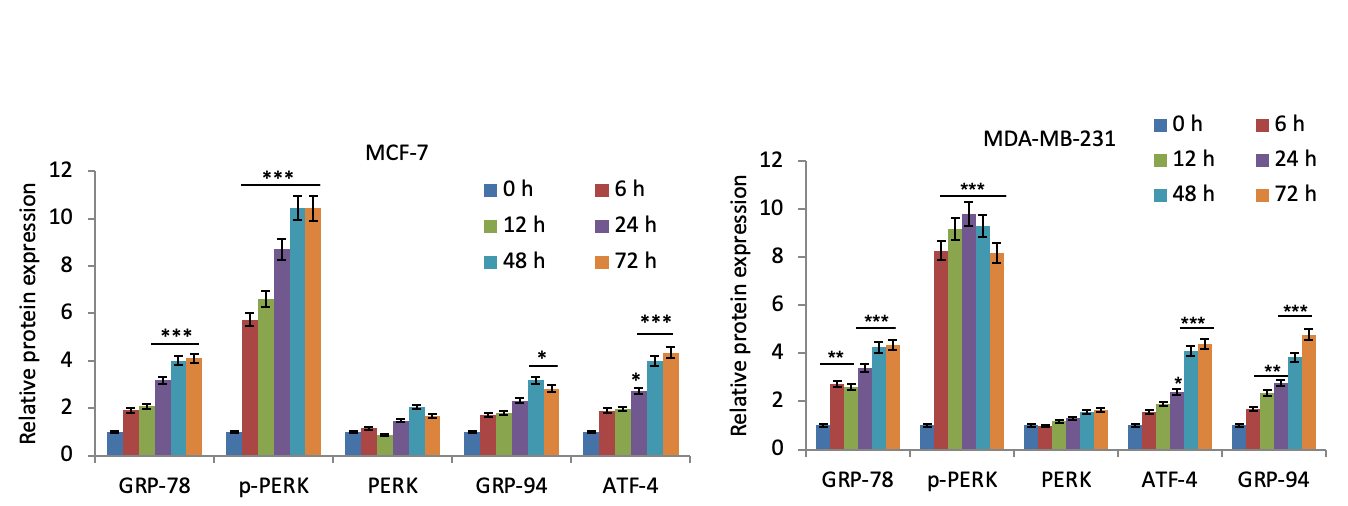


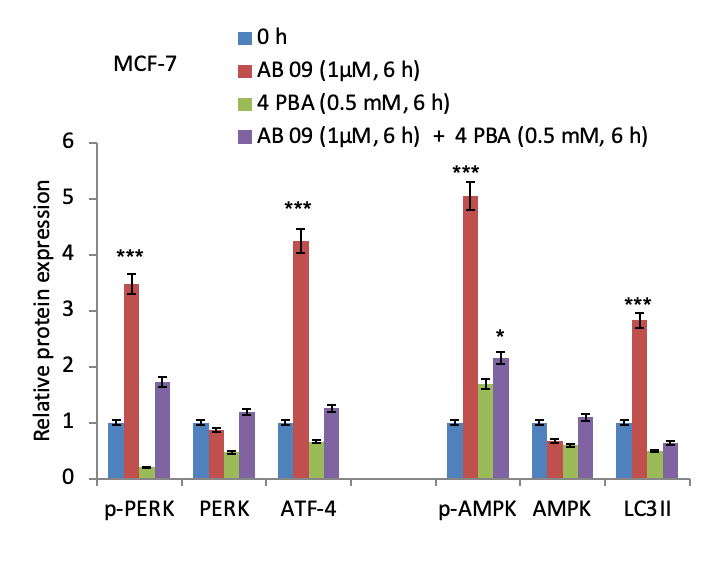


FIGURE 2


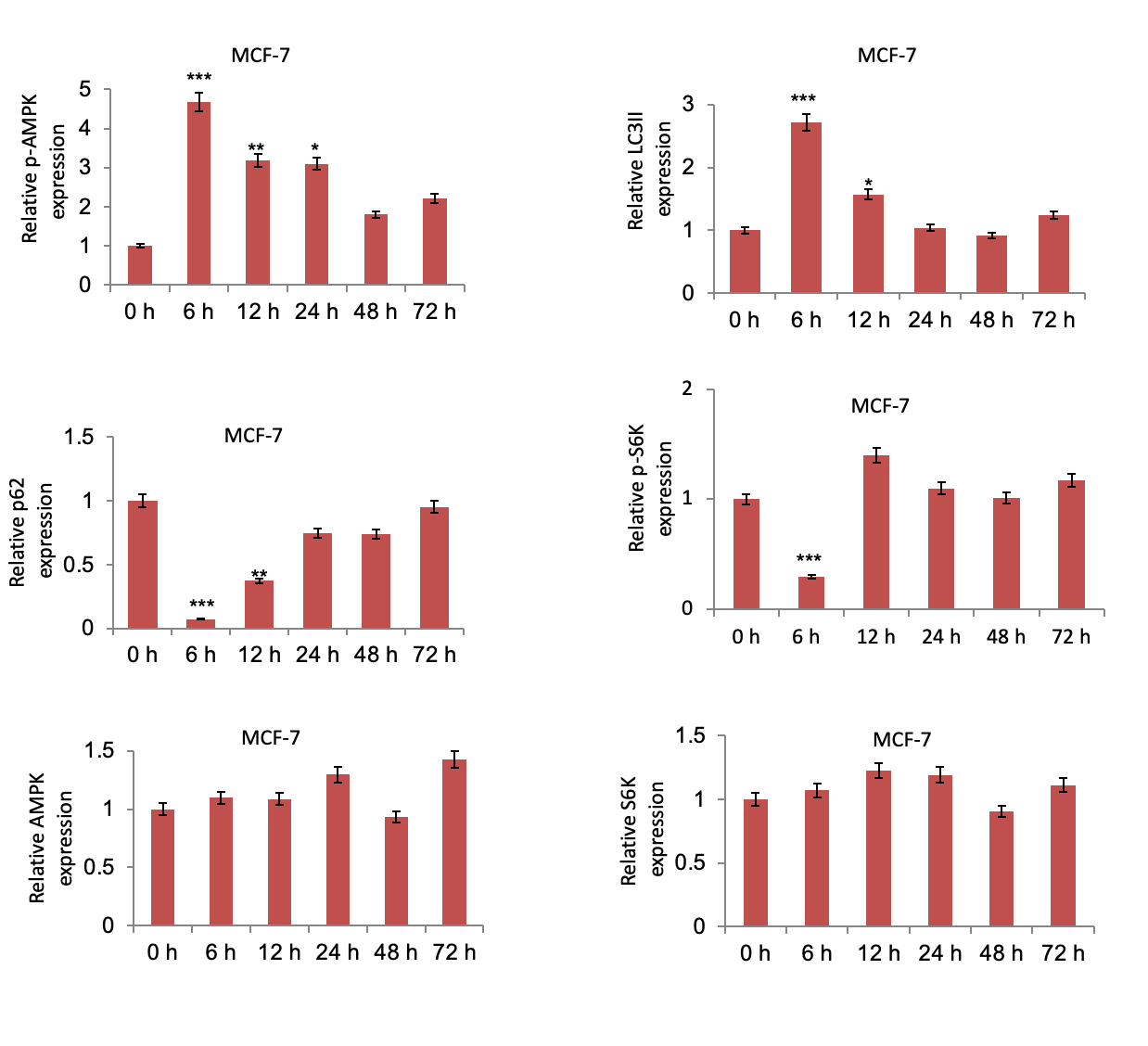


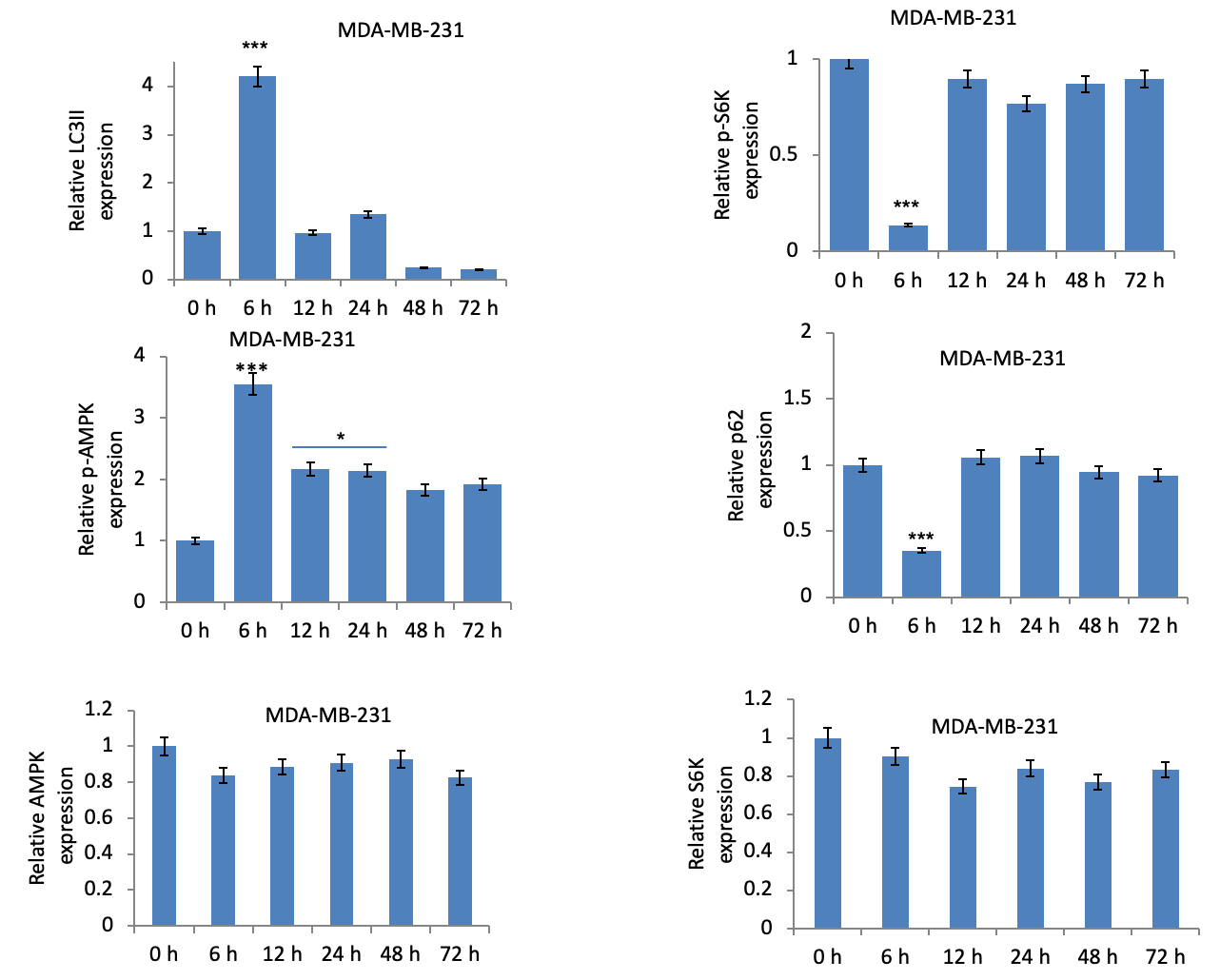


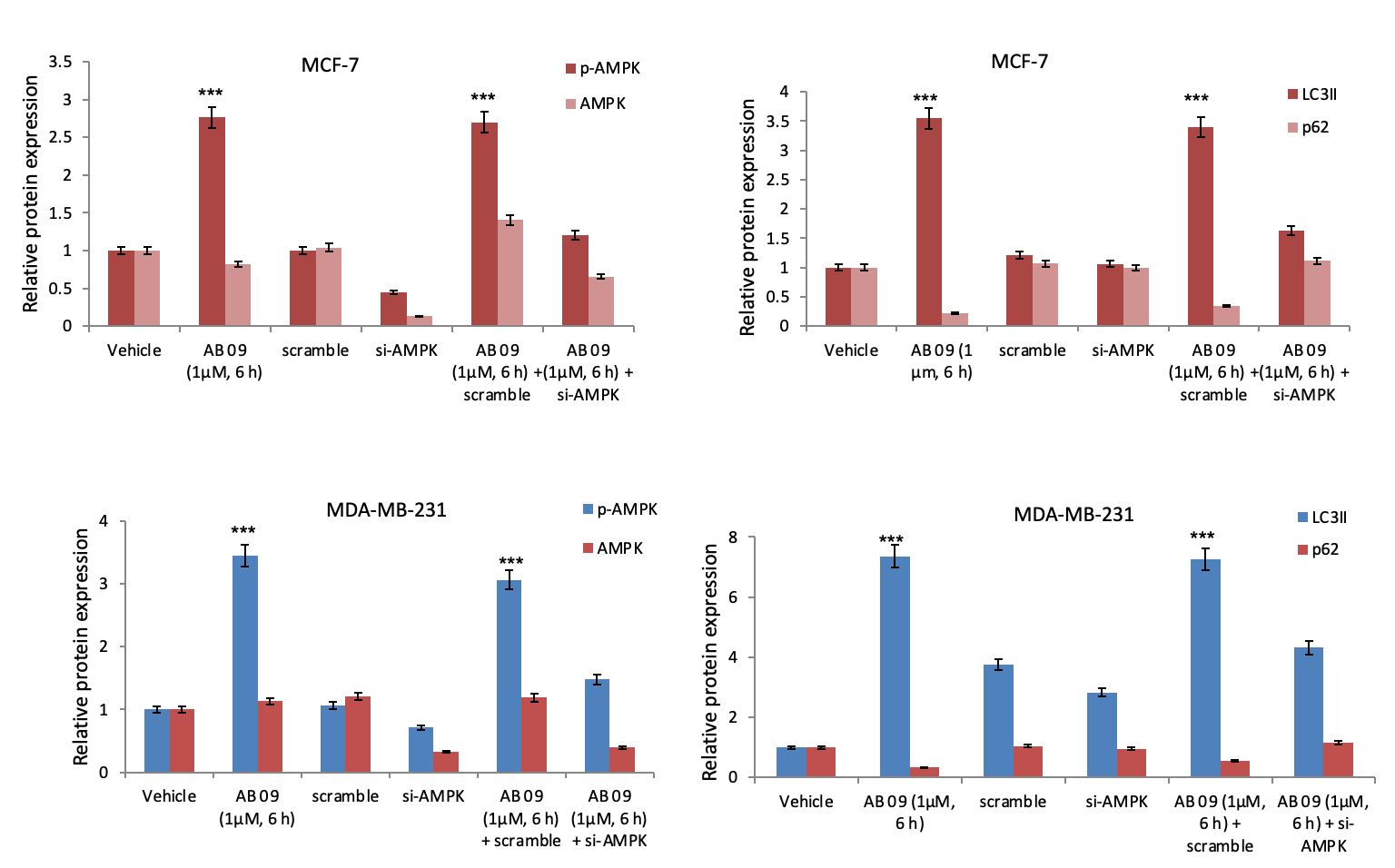


FIGURE 3


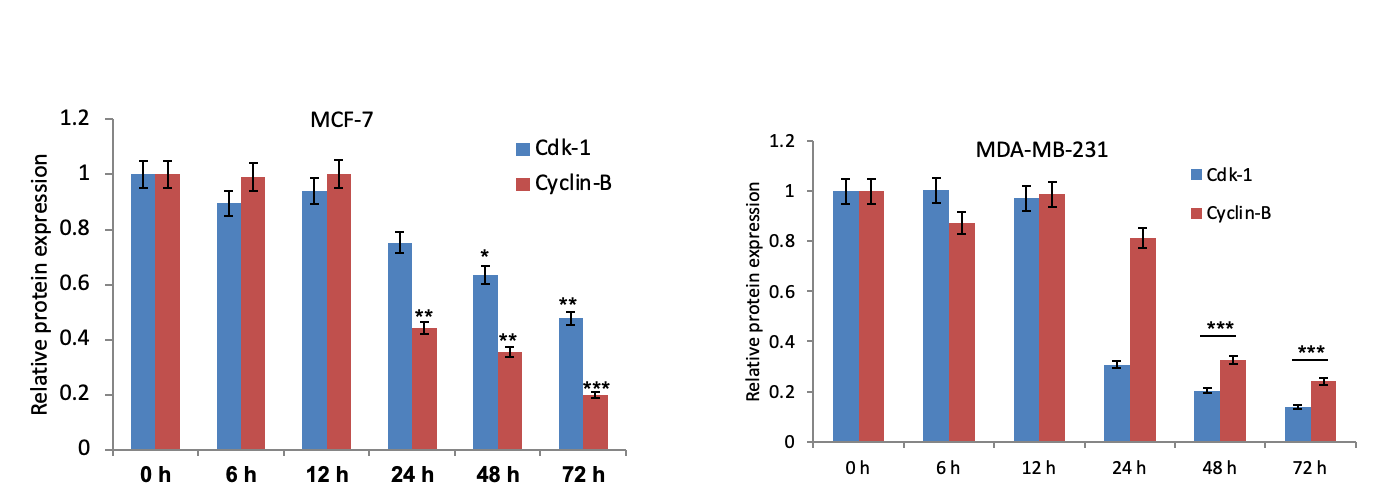


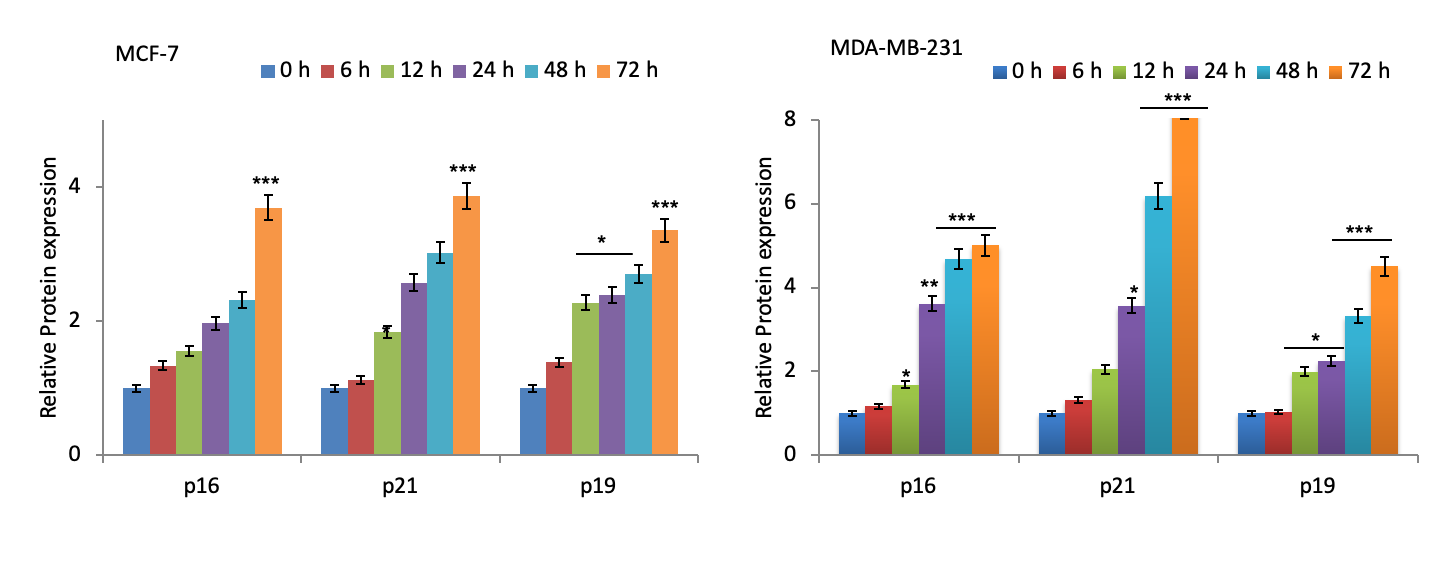


FIGURE 4
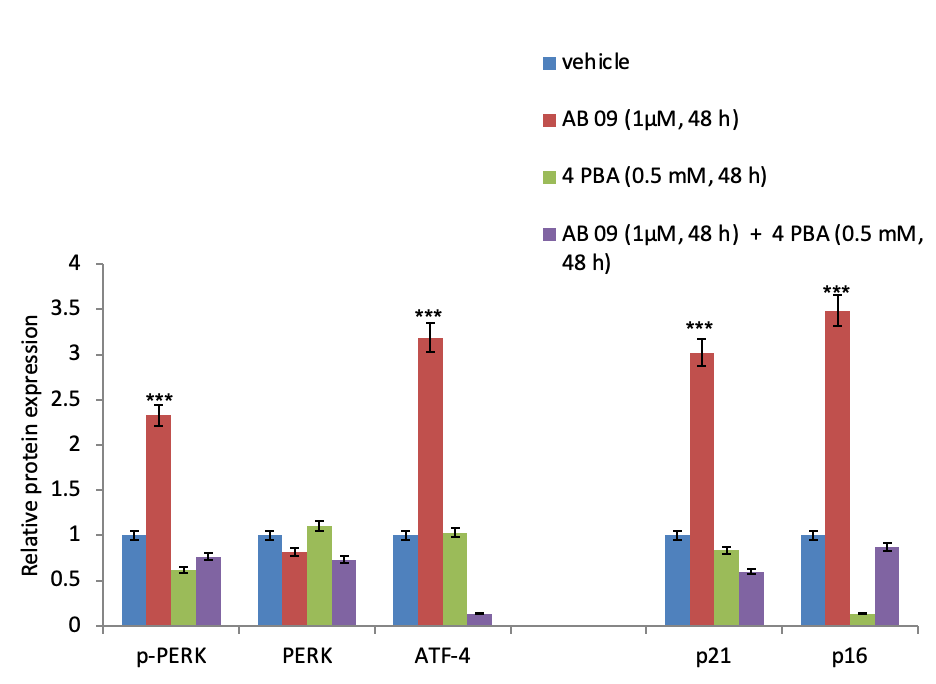

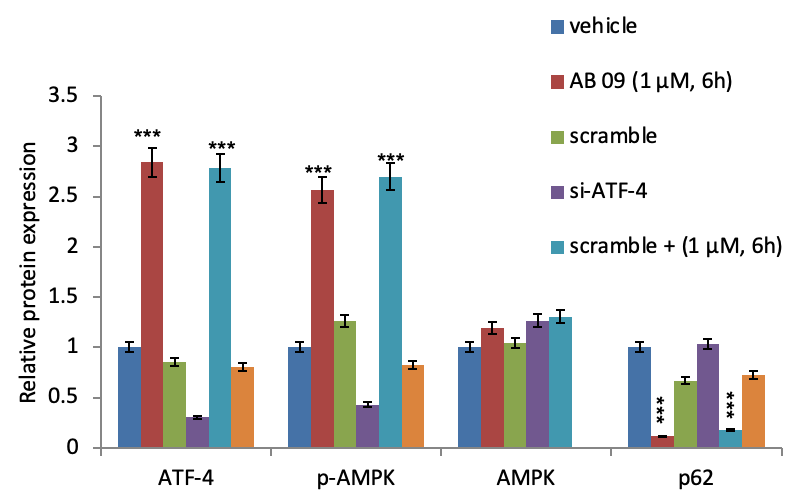

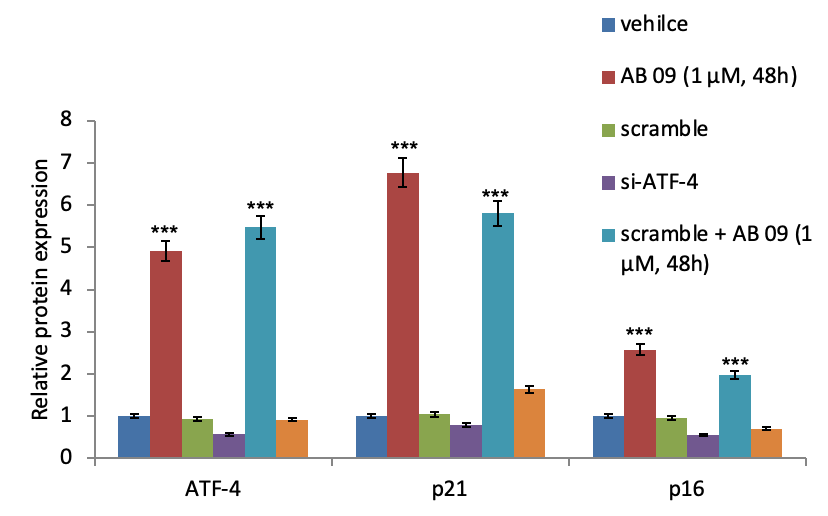


FIGURE 5


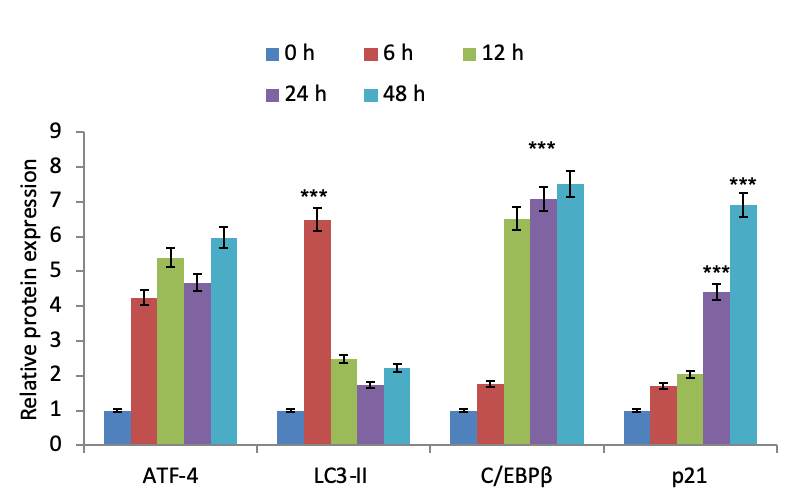


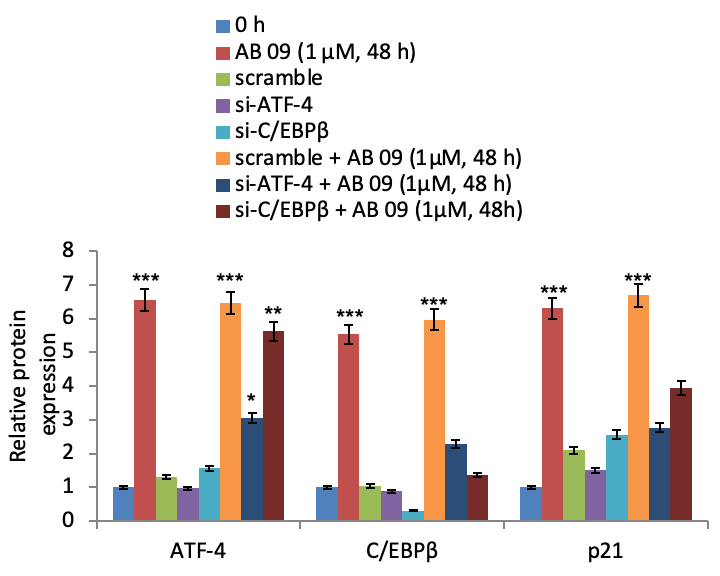


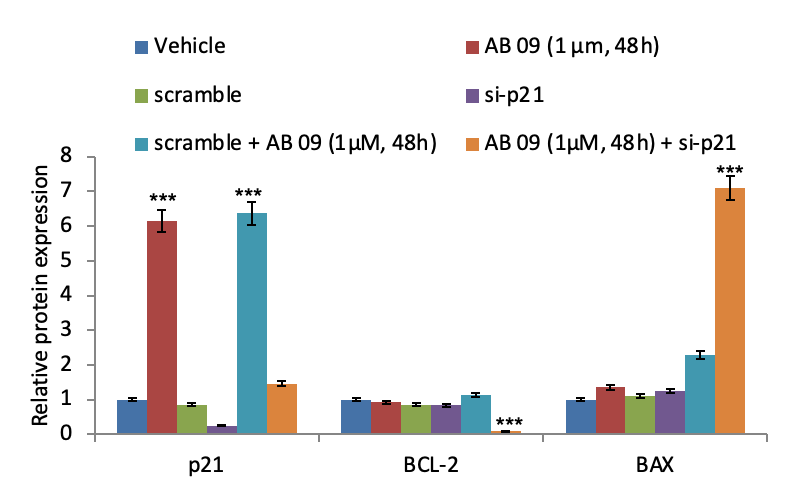


FIGURE 6


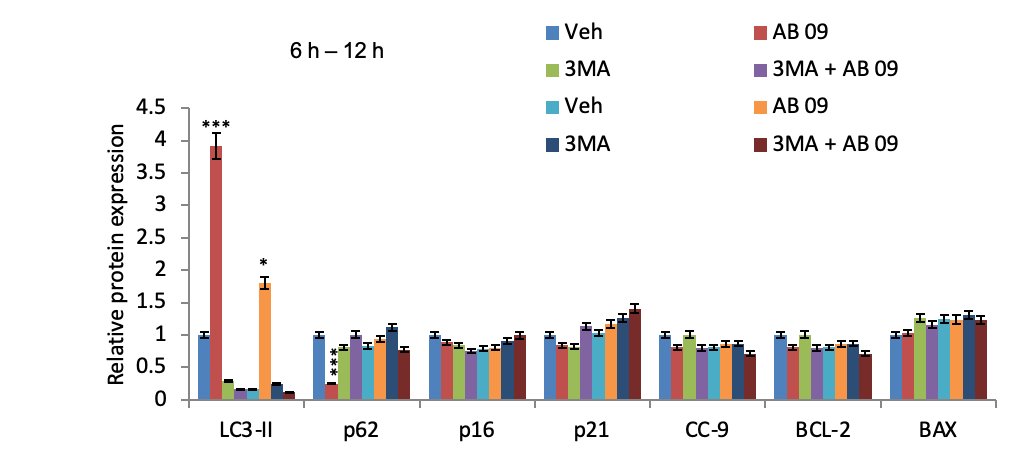


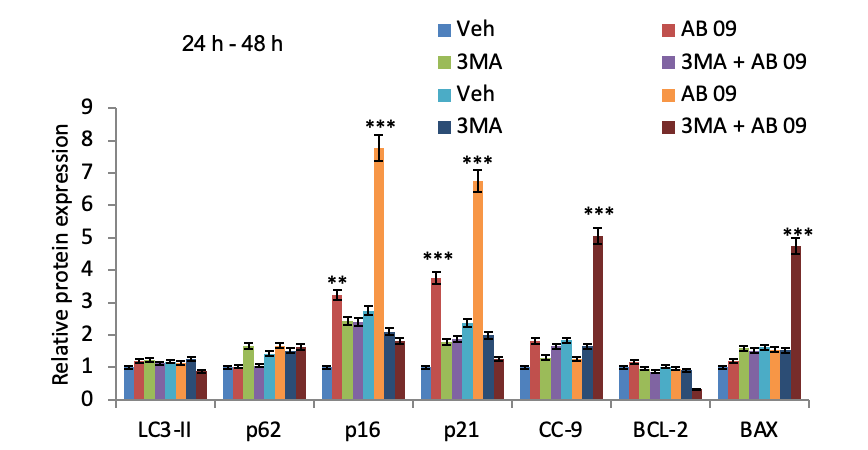


FIGURE 8


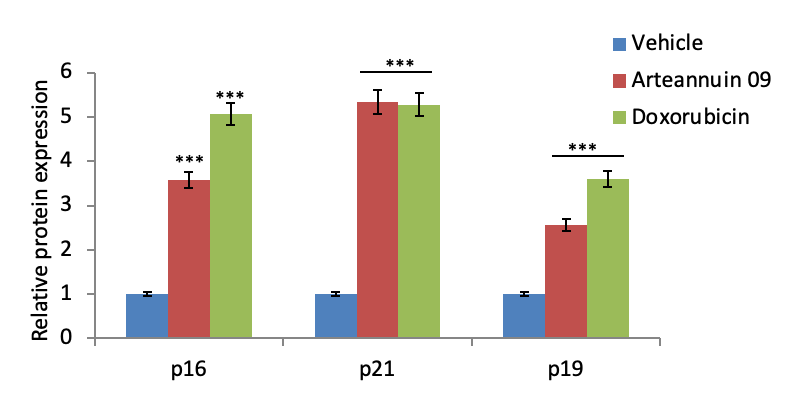


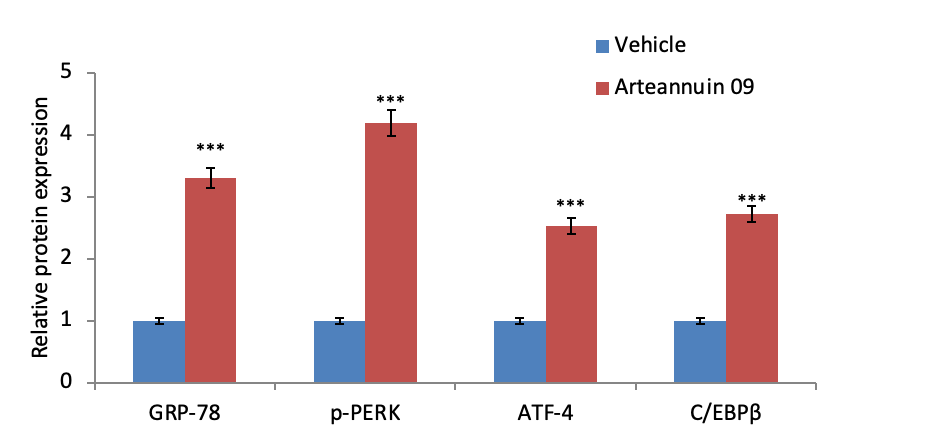


**3. List of antibodies**

**Supplementary Table 1**

| **S.no** | **Antibody** | **Application** | **Dilution** | **Source** |
| --- | --- | --- | --- | --- |
| 1 | Anti β-actin (A5316) mouse monoclonal  Beta-Actin (13E5) rabbit monoclonal | WB  WB | 1:2000  1:2000 | Sigma-Aldrich  Cell Signaling Technology |
| 2 | SQSTM1 (D-3) mouse monoclonal  SQSTM1/p62 (D5L7G) mouse monoclonal | WB  WB/ICC | 1:500  1:1000 | Santa Cruz Biotechnology  Cell Signaling Technology |
| 3 | P21(187) mouse monoclonal  P21 Waf1/Cip1 (12D1) rabbit monoclonal | WB  WB/ICC | 1:500  1:1000/1:200 | Santa Cruz Biotechnology  Cell Signaling Technology |
| 4 | Phospho-PRKAA/AMPKα (Thr172) (MA5-33216) rabbit monoclonal | WB/ICC | 1:1000/1:200 | ThermoFisher scientific |
| 5 | CDKN2A/p16INK4a(EPR1473) rabbit monoclonal | WB | 1:1000 | abcam |
| 6 | Anti-AMPKα1 (H-4): 398861 mouse monoclonal | WB | 1:500 | Santa Cruz Biotechnology |
| 7 | Atg5 (D5F5U) rabbit monoclonal | WB | 1:1000 | Cell Signaling Technology |
| 8 | Atg7 Antibody (2631) rabbit monoclonal | WB | 1:1000 | Cell Signaling Technology |
| 9 | p19 ARF (M-60) rabbit polyclonal | WB | 1:500 | Santa Cruz Biotechnology |
| 10 | Bax (2772S) rabbit monoclonal | WB | 1:1000 | Cell Signaling Technology |
| 11 | Cdk-1 (**MA5-11472**) mouse monoclonal | WB | 1:500 | ThermoFisher scientific |
| 12 | Cyclin –B (D-1) sc-166210 ) mouse monoclonal | WB | 1:500 | Santa Cruz Biotechnology |
| 13 | GRP-94 (H-212) rabbit polyclonal | WB | 1:500 | Santa Cruz Biotechnology |
| 14 | Anti-C/EBPβ (sc-7962) mouse monoclonal | WB | 1:500 | Santa Cruz Biotechnology |
| 15 | p53 (FL-393) rabbit polyclonal | WB | 1:500 | Santa Cruz Biotechnology |
| 16 | GADD 153 (F-168) rabbit polyclonal | WB | 1:500 | Santa Cruz Biotechnology |
| 17 | p-PERK (16F8) rabbit monoclonal | WB | 1:500 | Cell Signaling Technology |
| 18 | Cleaved Caspase 9 (D330) rabbit monoclonal | WB | 1:1000 | Cell Signaling Technology |
| 19 | ATF4(D4B8) rabbit monoclonal  ATF4 (22800) rabbit polyclonal | WB/ICC  WB | 1:1000  1:500 | Cell Signaling Technology  Santa Cruz Biotechnology |
| 20 | PERK (H-300) rabbit polyclonal | WB | 1:500 | Santa Cruz Biotechnology |
| 21 | Anti-rabbit IgG, HRP | WB | 1:2000 | Santa Cruz Biotechnology |
| 22 | Anti-mouse IgG, HRP | WB | 1:2000 | Santa Cruz Biotechnology |
| 23 | Alexa Fluor 488 F (ab’)2 fragment of goat anti-rabbit IgG (H+L) secondary antibody (A32727) | ICC | 1:500 | invitrogen |
| 25 | Bcl-2(N-19) rabbit polyclonal | WB | 1:500 | Santa Cruz Biotechnology |
| 26 | p70 S6 kinase (C -18) rabbit polyclonal | WB | 1:500 | Santa Cruz Biotechnology |
| 27 | Texas red^tm^ goat anti-rabbit IgG (H+L) secondary antibody (T2767) | ICC | 1:500 | invitrogen |
| 27 | P- p70 S6 kinase (T389) (108D2)  rabbit monoclonal | WB | 1:1000 | Cell Signaling Technology |
| 28 | LC3B (2775S) rabbit polyclonal  MAP LC3β (H-50) rabbit polyclonal | WB  WB | 1:1000  1:500 | Cell Signaling Technology  Santa Cruz Biotechnology |
| 29 | Beclin-1 (D40C5) rabbit monoclonal | WB | 1:1000 | Cell Signaling Technology |
| 30 | BiP (c50B12) rabbit monoclonal  GRP-78(H-129) rabbit polyclonal | WB  WB | 1:1000  1:500 | Cell Signaling Technology  Santa Cruz Biotechnology |
| 31 | p53 (1C12) Mouse mAb #2524S | WB | 1:1000 | Cell Signaling Technology |

WB, western blotting; ICC, Immunocytochemistry

**4. Reagents, chemicals and cell lines**

**Supplementary Table 2**

| **S. No** | **Name of chemical** | **Manufacturer** |
| --- | --- | --- |
| 1 | RPMI, DMEM, L-15 and FBS | Invitrogen |
| 2 | Trypsin-EDTA, | Sigma-Aldrich |
| 3 | Dithiothreitol (DTT), | Sigma-Aldrich |
| 4 | Doxorubicin,  Phenylmethylsulfonyl fluoride (PMSF), | Sigma-Aldrich |
| 5 | NP-40 | Sigma-Aldrich |
| 6 | Triton-X-100 | Sigma-Aldrich |
| 7 | Dimethyl sulfoxide (DMSO) | Sigma-Aldrich |
| 8 | penicillin/streptomycin | Sigma-Aldrich |
| 9 | Protease inhibitor cocktail (PI) | Sigma-Aldrich |
| 10 | Rapamycin | Sigma-Aldrich |
| 11 | Bradford’s reagent | Sigma-Aldrich |
| 12 | Bafilomycin | Sigma-Aldrich |
| 13 | 4-PBA | Selleck Chemicals, Houston, TX, USA |
| 14 | MCF-7(P-29) and MDA-MB-231(P-46) | American Type Culture Collection (ATCC, Manassas, VA, USA)/National Centre for Cell Science, Cell Repository. |

**5. siRNA sequences**

**Supplementary Table 3**

| S. no. | Gene name | Sequence |
| --- | --- | --- |
| 1 | PRKAA1 | Sense  GCAAUAAGCAUGCAUAAUAtt  Antisense  UAUUAUGCAUGCUUAUUGCtg |
| 2 | C/EBPβ | Sense  AGCACAGCGACGAGUACAAGAUCC  Antisense   CGGAUCUUGUACUCGUCGCUGUGCUUG |
| 3 | CDKN1A | Sense  CUU CGA CUU UGU CAC CGA G  Antisense  GAA GCU GAA ACA GUG GCU C |
